# Assessment of *in vitro* skin permeation and accumulation of phenolic acids from honey and honey-based pharmaceutical formulations

**DOI:** 10.1186/s12906-025-04786-1

**Published:** 2025-02-04

**Authors:** Anna Nowak, Anna Muzykiewicz-Szymańska, Magdalena Perużyńska, Edyta Kucharska, Łukasz Kucharski, Karolina Jakubczyk, Paulina Niedźwiedzka-Rystwej, Justyna Stefanowicz-Hajduk, Marek Droździk, Juraj Majtan

**Affiliations:** 1 Department of Cosmetic and Pharmaceutical Chemistry, Pomeranian Medical University in Szczecin, 72 Powstancow Wlkp. Ave, Szczecin, 70-111 Poland; 2 Department of Experimental and Clinical Pharmacology, Pomeranian Medical University in Szczecin, 72 Powstancow Wlkp. Ave, Szczecin, 70-111 Poland; 3 Department of Chemical Organic Technology and Polymeric Materials, West Pomeranian University of Technology in Szczecin, 10 Pulaski St, Szczecin, 70-322 Poland; 4 Department of Human Nutrition and Metabolomics, Pomeranian Medical University in Szczecin, 24 Broniewskiego St, Szczecin, 71-460 Poland; 5 Institute of Biology, University of Szczecin, 3c Felczaka St, Szczecin, 71-412 Poland; 6 Department of Biology and Pharmaceutical Botany, Medical University of Gdańsk, Al. Hallera 107, 80-416 Gdańsk, Poland; 7 Institute of Molecular Biology, Slovak Academy of Sciences, Dubravska cesta 21, Bratislava, 845 51 Slovakia; 8 Department of Microbiology, Faculty of Medicine, Slovak Medical University, Limbova 13, Bratislava, 833 03 Slovakia

**Keywords:** Honey, Polyphenols, Antioxidant activity, Wound healing, Biodegradation, Pharmaceuticals

## Abstract

**Background:**

Honey has been successfully used in wound care and cosmetics because of its effective biological properties, including antibacterial, antioxidant, and anti-inflammatory activities. Polyphenols, particularly phenolic acids, are key honey components responsible for these beneficial effects. In recent years, there has been a growing demand for natural, ecologically friendly, and biodegradable products in the modern cosmetics and wound care market. This study aimed to identify and quantify phenolic acids in four Polish honey samples of different botanical origins (heather, buckwheat, linden and rapeseed) and to assess for the first time the permeation of the identified phenolic acids through the skin and their accumulation after the application of pure honey samples, as well as honey-based hydrogel and emulsion formulations.

**Methods:**

The honey samples’ antioxidant activity and total phenolic content were determined using the DPPH and ABTS assays and the Folin–Ciocalteu method, respectively. Phenolic acids and volatile compounds were identified and quantified in honey samples using the HPLC-UV and GC-MS method, respectively. The biocompatibility of the honey samples was evaluated using a murine fibroblast cell line (L929). A Franz-type vertical diffusion cell with porcine skin was used to assess phenolic acid’s permeation and skin accumulation from different honey-based pharmaceutical formulations. The biodegradability of the prepared formulations was also characterised.

**Results:**

Gallic acid, 3,4-dihydroxybenzoic acid, 2,5-dihydroxybenzoic acid, coumaric acid, and 3-hydroxybenzoic acid were identified and quantified in the honey samples. Heather honey exhibited significantly higher antioxidant activity and total polyphenol content than the other honey samples. Heather, linden and buckwheat honey samples significantly decreased cell viability at concentrations of 5% and 2.5%, while rapeseed honey sample markedly reduced fibroblast viability only at 5%. Among the tested formulations - pure honey, hydrogel, and emulsion - higher skin permeation and accumulation rates of phenolic acids were observed with the prepared honey-based hydrogels than with the pure honeys and emulsions. Additionally, the prepared formulations were classified as partially biodegradable.

**Conclusions:**

The obtained results confirmed the effectiveness of two pharmaceutical formulations in the form of a hydrogel or emulsion containing honey after applied topically. The inclusion of honey in the vehicle, in particular hydrogel increased the penetration of phenolic acids through the skin.

**Supplementary Information:**

The online version contains supplementary material available at 10.1186/s12906-025-04786-1.

## Introduction

In the last years, there is a growing interest in cosmetic formulations that rely solely on natural components. This is related to, among other things, mitigate the negative impact of synthetic ingredients in cosmetics on natural environments, particularly aquatic fauna and flora [1].

Honeybee products are among the most frequently used natural ingredients in dermatology and skincare [2]. Furthermore, the positive clinical outcomes from topical application of honey have led to the development of ‘medical-grade honey’. Honey exhibits a range of biological activities, including antibacterial, antibiofilm, antioxidant, anti-inflammatory, and wound-healing properties. Among these, the antibacterial activity of honey is the most extensively studied and has recently been proposed as a complementary qualitative criterion for honey [3, 4]. The antibacterial properties of honey are mediated through the various substances and factors, including hydrogen peroxide, defensin-1, gluconic acid, low pH, and high osmolarity.

Honey is also used in wound healing and the treatment of dermatological disorders such as atopic dermatitis and psoriasis [5]. Manuka honey, topically applied to 14 patients with atopic dermatitis for 7 consecutive days, resulted in overall improvement of the disease [6]. It was shown that manuka honey significantly reduced the release of chemokine ligand (CCL) 26 (eotaxin 3), a major eosinophil chemoattractant to the site of inflammation. In addition, the inhibition of mast cell degranulation was observed after manuka honey treatment [6]. Another pilot study demonstrated the beneficial effects of kanuka honey in the treatment of psoriasis where high patient acceptability was observed when compared to an aqueous cream applied topically [7]. *In vitro* studies have shown that honey modulates several key pathophysiological processes in wound healing, including oxidative stress and inflammation. Honey and its polyphenolic components effectively counteract oxidative damage in skin and immune cells [8–12], helping to restore redox balance in wound sites accelerating the wound-healing process [13]. However, most experimental studies have used cell-based *in vitro* methods that do not fully capture the bioavailability and functionality of honey polyphenols in wound environments. To increase the beneficial properties of honey, it is often enriched with additional biologically active substances, such as vitamin C [14], fruit extracts [15], and algae [16].

Pharmacological activity of honey is determined by the content of various bioactive compounds, among others, phenolic acids, including benzoic acid derivatives (e.g., gallic acid (GA), 3,4-dihydroxybenzoic acid (3,4-DHB), 2,5-dihydroxybenzoic acid (2,5-DHB), 3-hydroxybenzoic acid (3-HB) and vanillic acid) and cinnamic acid derivatives (e.g., coumaric acid (CA), ferulic acid (FA) and caffeic acid). Phenolic acids and other antioxidants in honey are considered valuable therapeutic ingredients in pharmaceutical preparations. For example, GA, CA, FA and caffeic acid exhibit the strong anti-inflammatory and antioxidant activities [17, 18], and therefore, can be used for wound healing the treatment of various dermatological disorders and [19–21]. Furthermore, due to phenolic acids-mediated inhibition of melanin production, they can also be effective depigmenting or skin lightening agents [22]. Other phenolic acids such as 3,4-DHB or vanillic acid have also shown depigmenting effects [23]. Many previous studies have reported the potential anti-aging effects of phenolic acids. A 3,4-DHB induced the synthesis of collagen type I in human dermal fibroblast [24]; whereas CA and caffeic acid inhibited the activity of elastase, hyaluronidase and collagenase enzymes [25, 26].

The polyphenol content in honey, both in terms of quality and quantity, varies significantly depending on the honey’s botanical and geographical origin. Because of these variations, specific honey types (e.g., honeydew, manuka) are preferred for pharmaceutical and clinical use [27]. However, there are only a few reports in the literature on the cosmetic or dermatological use of honey of such type as heather, buckwheat, linden or rapeseed honey.

The combination of various polyphenols may create a synergistic effect, further increasing the therapeutic potential of honey-based formulations. However, to maximize such action, successful delivery of active substances through the skin layers is essential. The first step in this delivery process is the diffusion of polyphenols within the honey or honey formulations and their subsequent release from the matrix. Because of the complex and heterogeneous nature of skin structure, it is challenging to elucidate the dermal absorption of honey’s biologically active substances, including the permeation of honey polyphenols through the epidermis into the dermis. The Franz diffusion cell is the gold standard and most widely used method for assessing *in vitro* phenolic skin diffusion profiles with different skin models. The diffusion profiles of polyphenols, including flavonoids, vary due to differences in molecular weight, structure, and vehicle composition [28]. Components of the skin, such as the stratum corneum (SC) and sebum, often inhibit polyphenol permeation [28]. Moreover, the formulation type (e.g., emulsion, suspension, gel) significantly affects the dermal absorption of polyphenols. Understanding the penetration and accumulation of specific substances in the skin is critical for designing cosmetic or dermatological products containing honey. Phenolic acids have been shown to penetrate deeper skin layers, where they may exert antioxidant, anti-inflammatory, antibacterial, or anti-aging effects [29, 30].

Despite the honey is increasingly used as an active ingredient in dermatological or cosmetic preparations, characterisation of penetration potential of the key substances through the skin is missing. Therefore, the aim of our study was to identify and quantify selected phenolic acids of cosmetic importance in four Polish blossom honey samples of different botanical origin (buckwheat, heather, linden and rapeseed), as well as to assess honeys’ antioxidant activities. In the next stage, we attempted to assess the possibility of using the analyzed honeys in preparations applied to the skin, such as hydrogel and emulsion, and we also assessed, for the first time, the penetration of selected phenolic acids through pork skin and their accumulation in skin.

## Materials and methods

### Chemicals

2,2-Diphenyl-1-picrylhydrazyl (DPPH), 6-hydroxy-2,5,7,8-tetramethylchroman-2-carboxylic acid (Trolox), 2,2′-azino-bis(3-ethylbenzothiazoline-6-sulfonic acid) (ABTS), and hydroxyethyl cellulose were purchased from Sigma-Aldrich (St. Louis, MO, USA). Folin–Ciocalteu reagent, GA, 3,4-DHB, 3-HB, 2,5-DHB, CA, disodium phosphate, propylene glycol, and potassium dihydrogen phosphate were obtained from Merck (Darmstadt, Germany). Acetic acid, aluminium chloride, hydrochloric acid, sodium sulphate anhydrous, sodium lauryl sulphate, phosphate-buffered saline (PBS), propylene glycol, ethanol, methanol, and isopropanol were sourced from Chempur (Piekary Śląskie, Poland). Acetonitrile for high-performance liquid chromatography (HPLC) was purchased from J.T.Baker (the Netherlands). Sodium dodecyl sulphate (SDS), 99.0% (Sigma-Aldrich) was used as a reference standard and positive control in biodegradation studies [31]. All reagents were of analytical grade.

### Honey samples

Four monofloral honey samples certified as “Miód drahimski” were used in this study: heather honey (*Calluna vulgaris* (L.) Hull) (HH), buckwheat honey (*Fagopyrum esculentum* Mill.) (BH), rapeseed honey (*Brassica napus* var. *arvensis*) (RH), and linden honey (*Tilia cordata* Mill.) (LH). These samples were provided by a certified beekeeper from Poland and samples fulfil all criteria according to Official Journal of European Union (C248/8) for “Miód drahimski”, including criteria for percentage of dominant pollen: > 45% buckwheat pollen (Fagopyrum), > 45% heather pollen (*Calluna vulgaris*), > 45% colza pollen (*Brassica napus* var. Arvensis), > 20% linden pollen (Tilia) and < 35% proportion of any plant pollen. The certification of all honey samples was carried out by BioCert Małopolska Sp. z o (Poland).

The honey samples were collected from the Drawsko Landscape Park area (53°40′N, 16°10′E), and their botanical origins were determined by the trained beekeeper based on the availability of flora for nectar foraging, the location of the apiary, and the organoleptic characteristics of the honey. The samples were deposited in the plant material storage room at the Chair and Department of Cosmetic and Pharmaceutical Chemistry of the Pomeranian Medical University (No. H-AM2024-01). Honey samples were stored in accordance with the Polish Standard PN-88/A-77,626. Honey samples were stored in a dry and clean room, sufficiently airy, free from foreign odours and pests, protected against access by insects (bees, wasps, flies, etc.). The storage temperature was no higher than 18⁰C. Samples were stored in safe glass packaging, protected from access to light.

### Identification and quantification of phenolic acids in honey samples

For HPLC analysis, the honey samples were diluted with distilled water at 25 °C in proportions of 1:10 until completely dissolved. The samples were mixed on a magnetic stirrer for 10 min, centrifuged, and subjected to HPLC analysis to determine the content of individual phenolic acids. The identification and quantification of phenolic acids in the honey samples were carried out using an HPLC system from Knauer (Berlin, Germany) coupled with the WellChrom UV K-2600 detector. The tested components were separated on a 125- × 4-mm column containing Hypersil ODS with a particle size of 5 μm. The mobile phase consisted of acetonitrile, 1% acetic acid, and methanol (47.5:47.5:5.0 by volume), and the flow rate was 1 mL/min. Twenty µL of the sample (honey solutions) were injected into the column. The same chromatographic conditions were used fort the standard substances and honey samples. The phenolic acids were identified based on their individual standards retention times at wavelength of 280 nm. To prepare calibration curves, the following concentrations’ range was used for all standards: 0.0031, 0.0063, 0.0125, 0.0250, 0.0500 and 0.1000 mg/mL. The correlation coefficient of the calibration curve was 0.9999 for GA (y = 30074x − 1.1923, Rt = 5.962 min), 0.9999 for 3,4-DHB (y = 20740 − 0.5806, Rt = 12.042 min), 0.9997 for 2,5-DHB (y = 31388x + 0.2252, Rt = 14.274 min), 0.9998 for CA (y = 39902x − 2.2879, Rt = 18.022 min), and 1.0000 for 3-HB (y = 16798x + 0.2776, Rt = 24.092 min). All samples were analysed three times.

### Determination of volatile compounds of nectar honeys

A supernatant phase analysis technique was used to extract the volatile compounds contained in the tested honey samples. The determination was performed as follows: 5 g of suitable honey was weighed into a 15 mL glass vial. The tightly closed vial was heated at 60 °C. for 60 min. After this time, 1 mL of the supernatant phase was collected and analysed by a gas chromatography coupled to mass spectrometry (GC-MS). GC-MS analysis was performed using a Shimadzu GC-MS-QP2020 NX (Shimadzu, San Jose, CA, United States) with a Shimadzu SH-I-5MS column (30 m × 0.25 mm × 0.25 μm). The column temperature was kept at 40 °C for 2 min and programmed to 300 °C at a rate of 10 °C/min and kept constant at 300 °C for 2 min. Helium’s flow rate as a carrier gas is 35 cm/s (1 µL/min). MS were taken at 70 eV with split 10. The analysis duration was 30 min, and the sample volume was 1 mL. Identification of the constituents of the supersurface phase of honey samples was made by comparison of their mass spectra located in the spectra library (NIST-2020). All samples were tested three times.

### Determination of antioxidant activity and total phenolic content of honey samples

The scavenging activity of DPPH stable free radicals in the analysed honey samples was measured as previously described [32]. Briefly, a 0.15-mL aliquot of each sample was mixed with 2.85 mL of 0.3 mM DPPH radical solution dissolved in 96% (v/v) ethanol. The absorbance of the DPPH working solution was adjusted to 1.00 ± 0.02 at 517 nm using 70% (v/v) ethanol. After 10 min of incubation in the dark at room temperature, the absorbance at 517 nm was measured against 70% (v/v) ethanol using a Hitachi UV-Vis Spectrophotometer U-5100 (Tokyo, Japan).

The ABTS radical scavenging activity was evaluated as previously described [29]. A stock solution was prepared by dissolving 7 mmol/L ABTS in a 2.45 mmol/L aqueous potassium persulfate solution. The solution was incubated for 24 h in the dark at room temperature and then diluted with 50% (v/v) methanol to create a working solution. A 2.5-mL aliquot of the ABTS working solution was mixed with 0.025 mL of the analysed hydrogels in a spectrophotometric cuvette. After 6 min of incubation in the dark at room temperature, the absorbance was measured at 734 nm.

The scavenging activity on the DPPH and ABTS radicals was expressed as a percentage of inhibition using the following equation:

$$\% DPPH\left( {ABTS} \right)scavenging = 1 - As/\,Ac \times 100\% ,$$

where:

As = absorbance of the tested sample.

Ac = absorbance of the control sample.

Measurements were carried out in triplicate for each honey sample.

In comparison, as a reference, 6-hydroxy-2,5,7,8-tetramethylchroman-2-carboxylic acid (trolox) was used, and the antioxidant activity of honeys as well as individual phenolic acids were carried out. The results are presented as trolox equivalents in mmol trolox/dm^3^.

As previously described, total polyphenol content (TPC) in the analysed honey samples and acceptor fluids was determined using the Folin–Ciocalteu method [33], . Briefly, 0.15 mL of 10-fold diluted Folin–Ciocalteu reagent, 1.35 mL of 0.01 M sodium carbonate solution, and 1.35 mL of distilled water were mixed with 0.15 mL of the sample. The cuvette was sealed with a stopper and incubated for 15 min at room temperature. The spectrophotometric measurement was then performed at 765 nm. GA was used as a standard, and the results were expressed as mg GA/100 g of honey. Three independent measurements were conducted.

### Cell viability assay

The biocompatibility study was conducted using a murine fibroblast cell line (L929). Cell viability was evaluated using the resazurin-based PrestoBlue™ HS Cell Viability Reagent (Thermo Fisher Scientific, Waltham, MA, USA). The assay principle is based on viable cells continuously converting resazurin into highly fluorescent resorufin, with the amount of resorufin directly correlating to the number of metabolically active cells. Cells were seeded in 96-well black microplates (Greiner, Austria) at a density of 5 × 10^3^ cells/well and cultured in DMEM high-glucose medium (Sigma-Aldrich Merck Group, USA) supplemented with 10% heat-inactivated fetal bovine serum (EURx, Poland), 2 mM L-glutamine (Sigma-Aldrich Merck Group), and penicillin-streptomycin (Sigma-Aldrich Merck Group). After 24 h, the cell culture medium was removed and replaced with 100 µL of fresh medium containing 0.01%, 0.1%, 0.2%, 0.5%, 1%, 2.5%, and 5% honey. The 5% (w/v) honey solutions were prepared in a complete cell culture medium, sterilised using 0.22-µm membrane filters, and diluted in a complete cell culture medium as described above. Cells cultured with medium alone were used as the negative control.

After 24 h of treatment, the honey solutions/medium were replaced with fresh medium (90 µL), and 10 µL of PrestoBlue reagent was added to each well and incubated for 30 min under standard cell culture conditions (5% CO_2_ and 37 °C). Fluorescence was measured using a spectrophotometric microplate reader (Infinite 200 Pro; Tecan, Switzerland) at excitation/emission wavelengths of 560/594 nm. Results were normalised to the negative control (100% viability) and obtained from at least three independent experiments, each conducted in triplicate. The IC_50_ values were evaluated using an online calculator (AAT Bioquest, Inc., Quest Graph™ IC_50_ Calculator (v.1); retrieved from https://www.aatbio.com/tools/ic50-calculator-v1, accessed on 19 June 2024).

Under the same conditions, after 24 h of treatment, the CytoTox 96 Non-Radioactive Cytotoxicity Assay (Promega, USA) was used to evaluate lactate dehydrogenase (LDH) leakage. The release of intracellular LDH into the culture medium indicates irreversible cell death resulting from cell membrane damage. Following the manufacturer’s protocol, 50-µL aliquots from all test and control wells were transferred into fresh 96-well flat clear bottom plates. The reconstituted substrate mix (50 µL) was added to each well, and the plates were incubated at room temperature for 30 min, protected from light. Finally, 50 µL of stock solution was added to each well, and absorbance was measured at 490 nm using the spectrophotometric reader (Infinite 200 Pro; Tecan). Untreated cells served as the negative control, while cells treated for 30 min with lysis solution (0.9% Triton X-100) were used as the positive control (maximum LDH release). The readings were obtained from three independent experiments, each conducted in triplicate. The percentage of cytotoxicity was calculated using the formula:

$$\begin{gathered}Viability\,\left( \% \right) = \,100 - (experimental{\text{ }}LDH{\text{ }}release - negative{\text{ }}control) \hfill \\\,\,\,\,\,\,\,\,\,\,\,\,\,\,\,\,\,\,\,\,\,\,\,\,\,\,\,\,\,{\text{ /}}(maximum{\text{ }}LDH{\text{ }}release - negative{\text{ }}control) \times 100\% \hfill \\ \end{gathered}$$

Additionally, optical microscopy imaging of L929 cells after 24 h of treatment was performed using a Smart Fluorescent Cell Analyzer Microscope JuLi (Korea), limited to the negative and positive controls and the highest honey concentrations (5%, 2.5%, and 1%).

### Hydrogels and emulsion preparations

**Fig. 1:**
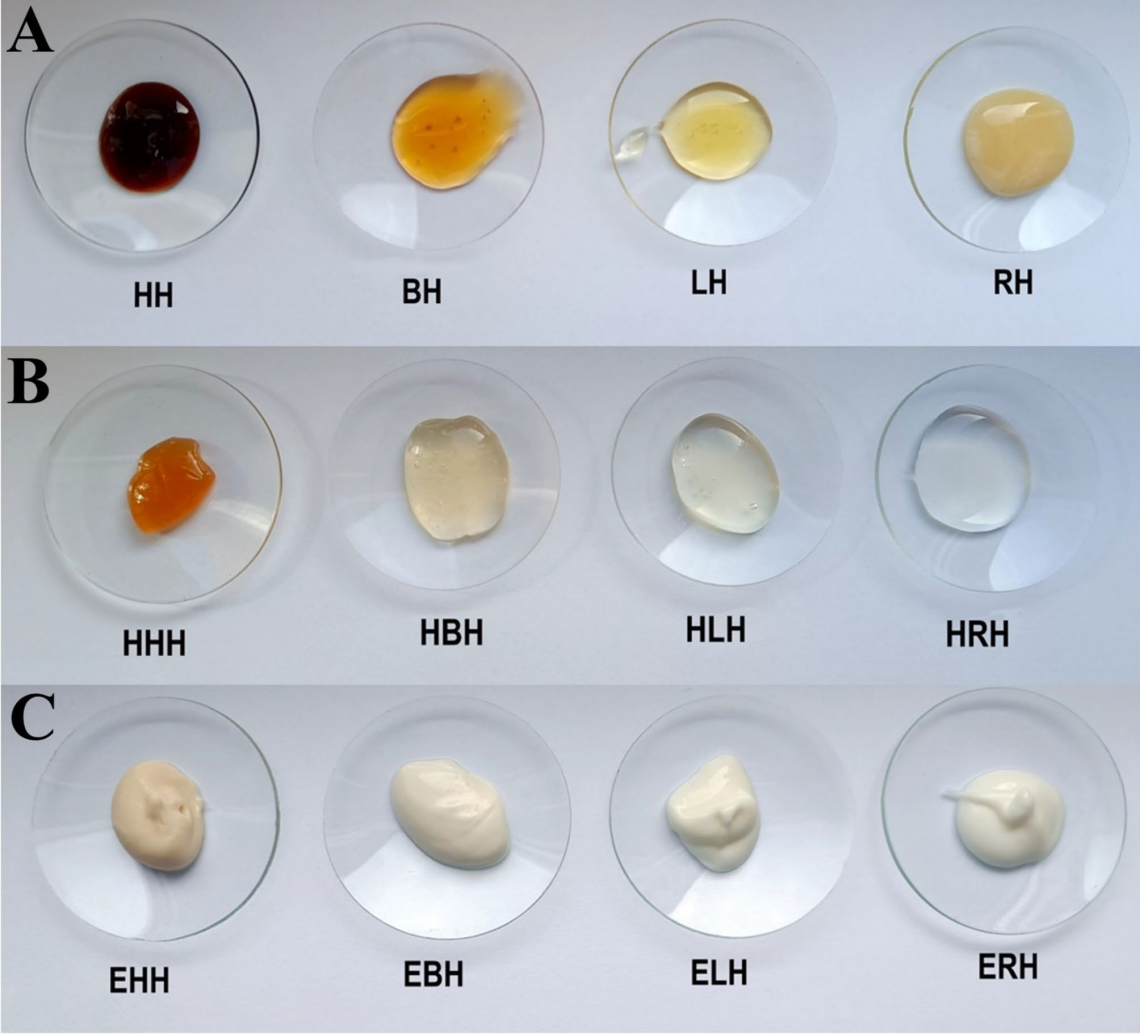
Honey samples and their pharmaceutical formulations used in the study. **(A)** Pure honey samples; **(B)** Honey-based hydrogels; **(C)** Honey-based emulsions. HH: heather honey; BH: buckwheat honey; LH: linden honey; RH: rapeseed honey; HHH: hydrogel with HH; HBH: hydrogel with BH; HLH: hydrogel with LH; HRH: hydrogel with RH; EHH: emulsion with HH; EBH: emulsion with BH; ELH: emulsion with LH; ERH: emulsion with RH

The ingredients used to prepare the substrates are presented in Table 1. To prepare the hydrogel, honey was dissolved in water by stirring on a magnetic stirrer with a heating function set to 35 °C. Once the honey was completely dissolved, glycerine and hydroxyethyl cellulose were added. Mixing continued until a uniform and clear gel was formed (Fig. 1B).

**Table 1:** Composition of hydrogels and emulsions containing honey samples used in the study

	Honey-based formulations	
Hydrogel	Emulsion (oil-in-water)	
	water phase	oil phase
10% honey 5% glycerine 5% hydroxyethylcellulose (HEC) 80% distilled water	10% honey 5% glycerine 52% distilled water	20% grape seed oil 7% beeswax (bleached) 6% Biobaza^®^ (Glyceryl Stearate, Cetearyl Alcohol, Sodium Stearoyl Lactylate)

For emulsion preparation, the ingredients of the oil phase and the water phase (except for the honey) were weighed in separate beakers and heated to 70 °C. After removing the aqueous phase from the water bath, the honey was dissolved in it. The oil phase was then slowly poured into the water phase while stirring intensively with a mixer until the mixture cooled completely and reached a uniform consistency (Fig. 1C).

### Stability of honey-based hydrogels and emulsions

The stability of all honey-based hydrogels and emulsions was tested according to a modified method by Muthachan and Tewtrakul [34]. The separation of the preparations was evaluated using a centrifuge test. Preparation samples (3 g) were centrifuged (MPW-223e; Mechanika Precyzyjna, Warsaw, Poland) at 4000 rpm and 25 °C for 10 min to assess the potential for phase separation.

Additionally, the stability of all preparations was evaluated using a heating-cooling test. The samples were incubated at 45 °C in a drying oven (DHG-9075 A) for 48 h, followed by incubation at 4 °C for 48 h. This test was conducted over six cycles. After each cycle, the preparations were inspected visually for any changes in appearance.

### *In vitro* skin permeation testing

In the *in vitro* permeation experiments, porcine abdomen skin was used because of its similar permeability to human skin [35, 36]. The skin for the experiment was prepared and stored as described previously [33, 37]. The skin was obtained from a local slaughterhouse. First, fresh skin was washed several times in PBS buffer pH 7.4 and skin fragments of 0.5 mm thickness was subsequently dermatomized. Next, it was divided into 2 cm x 2 cm pieces, wrapped in aluminium foil and stored in a freezer at − 20 °C until use. Skin samples were stored for no longer than three months. On the day of the experiment, skin samples were slowly thawed at room temperature for 30 min. The study was conducted in compliance with ethical principles.

Permeation experiments were conducted using Franz diffusion cells (SES GmbH Analyse Systeme, Bechenheim, Germany) with a diffusion area of 1 cm^2^. The acceptor chamber was filled with PBS solution (pH 7.4), and a constant temperature of 37.0 °C ± 0.5 °C was maintained in each diffusion unit. The content of the acceptor chamber was stirred with a magnetic stirring bar at a consistent speed across all cells. The donor chamber had a volume of 2 mL, while the acceptor chamber had a volume of 10 mL.

Undamaged pieces of skin were placed between the donor and acceptor chambers of the Franz diffusion cells. Skin integrity was measured using an LCR meter 4080 (Voltcraft LCR 4080; Conrad Electronic, Germany) according to previous studies [29, 37]. Next, 1 g of each honey sample or their prepared formulations (Table 1) was applied to the outer side of the skin in the donor compartment. The penetration study was carried out over 24 h. At 3, 5, and 24 h, 0.5-mL samples were withdrawn from the acceptor chamber and replaced with the same volume of fresh PBS. The concentrations of phenolic acids in the acceptor phase were evaluated using HPLC. The cumulative mass (µg) of each phenolic acid was calculated based on the obtained concentrations.

### Accumulation of phenolic acids in the skin

The assessment of phenolic acid accumulation in the skin was conducted similarly to our previous study [30]. Briefly, after the permeation experiment, the skin was cut around the diffusion area (1 cm^2^) and incubated in 2 mL of methanol for 24 h. Following incubation, the skin samples were homogenised using a homogeniser (IKA^®^ T18 digital ULTRA-TURRAX; Staufen, Germany). The supernatant was collected for HPLC analysis.

The accumulation of phenolic acids in the skin was calculated by dividing the amount of each substance remaining in the skin by the mass of the skin sample. Results were expressed as the mass of phenolic acid per mass of skin (µg/skin).

### Biodegradation studies

The biodegradation of all honey-based hydrogels and emulsions was conducted according to a methodology recommended by the Organisation for Economic Cooperation and Development (OECD), which is based on a general method of measuring aerobic biodegradability in a mineral environment through CO_2_ production [38]. The concentration of suspended solids and the total number of microorganisms (CFU/mL) in activated sludge, collected from the aeration chamber of the Pomorzany wastewater treatment plant in Szczecin, were determined using a microbiological test (Schülke Mikrocount Duo) with TTC medium and agar containing Tergitol-7. The test was immersed in activated sludge for 10 s, and the number of bacteria was assessed by comparing the appearance of the resulting test with the reference test after 96 h at room temperature (Figure S1). The procedure for the biodegradation of cosmetic preparations was described in a recent study [39], and the system for measuring CO_2_ produced by microorganisms during the 28-day test is presented in the supplementary material (Figure S2).

The only carbon and energy sources were the tested formulations and SDS, which served as a reference compound at concentrations of 40 mg/L organic carbon. The carbon content of the tested formulations and the reference compound was determined by CHNS/O elemental analysis using a FLASH 2000 CHNS/O analyser (Thermo Fisher Scientific). Samples were weighed in tin crucibles (2.4–2.8 mg) to the nearest 0.001 mg. The device was calibrated using L-methionine, sulfanilamide, 2,5-(bis(5-tert-butyl-2-benzoxazol-2-yl) thiophene (BBOT), and L-cysteine as standards [39]. The initial concentrations for the formulations and the reference compound were as follows: HHH = 348 mg/L, HBH = 424 mg/L, HLH = 580 mg/L, HRH = 468 mg/L, EHH = 88 mg/L, EBH = 96 mg/L, ELH = 112 mg/L, ERH = 76 mg/L, and SDS = 81 mg/L.

Biodegradation tests for each formulation were conducted in three test vessels. The quantity of CO_2_ generated was calculated using total organic carbon analysis (TOC-LCSH/CSN; Shimadzu Corporation). The inorganic carbon content of the test specimens was calculated using the calibration curve y = 4.1187x + 7.1718, R^2^ = 0.999. The degree of biodegradation of the test formulations was calculated using the following formula:

$$\% B = \frac{{\left[ {{C_{ICi}} \cdot \:{V_0} + \sum\nolimits_{i = 1}^n {\left( {{C_{ICi + 1}} + {C_{ICi}}} \right)} \cdot \:\left( {{V_0} - i \cdot \:{V_p}} \right)} \right] \cdot \:R}}{{m \cdot \:U}} \cdot \:100\%$$

where:

*%B* = degree of biodegradation.

*C*_*IC*_ = concentration of inorganic carbon in test vessel 1.4, obtained by total organic carbon analysis of the test sample corrected by blank (mg/L).

*R* = dilution of the sample collected from test vessel 1.4 (2.5).

*V*_*0*_ = initial volume of NaOH solution in test vessel 1.4 (0.25 L).

*i* = sample number.

*V*_*p*_ = volume of sample taken from test vessel 1.4 (0.01 L).

*m* = mass of test formulation injected into test vessel 1.3 (mg).

*U* = proportion of carbon in the test formulation introduced into test vessel 1.3.

### Statistical analysis

Results are presented as the mean ± standard deviation (SD) and the relative standard deviation (RSD). One-way analysis of variance (ANOVA) was performed to assess the data, and Tukey’s test was used to evaluate the significance of differences between individual groups (α = 0.05). Pearson correlation was also performed to assess the relationship between DPPH or ABTS methods and TPC. Statistical differences between control and treated cells during cell viability assays were evaluated using Student’s t-test. Statistical calculations were conducted using Statistica 13 PL software (StatSoft, Krakow, Poland).

## Results

### HPLC analysis of honey phenolic acids

The content of selected phenolic acids in the honey samples is presented in Table 2. The identified and quantified phenolic acids include GA, 3,4-DHB, 2,5-DHB, CA and 3-HB (Figure S3). The content of phenolic acids varied among the honey samples. Heather honey had the highest significant content of GA (2.79 ± 0.08 mg/100 g of honey), while linden honey had the lowest (0.34 ± 0.01 mg/100 g of honey). On the other hand, linden honey contained significantly the highest amount of 2,5-DHB (0.54 ± 0.02 mg/100 g honey) in compared to other honeys. Heather honey was also characterized by the highest content of CA (0.24 ± 0.01 mg/100 g of honey) and together with linden honey, it had the highest content of 3-HB (0.18 ± 0.01 and 0.17 ± 0.02 mg/100 g of honey, respectively).

**Table 2:** Content of identified phenolic acids in analysed honey samples (*n* = 3). The data are expressed as mean values ± standard deviation (SD) and relative standard deviation (% RSD). One-way ANOVA followed by Tukey’s post hoc test; different letters also mean significant differences between individual honeys, α = 0.05

Phenolic acid	Honey sample			
	HH	BH	RH	LH
	mg/100 g of honey (%RSD)			
GA	2.79 ± 0.08^c^ (1.29%)	0.99 ± 0.02^b^ (0.70%)	0.98 ± 0.03^b^ (2.70%)	0.34 ± 0.01^a^ (4.23%)
3,4-DHB	0.35 ± 0.03^b^ (2.49%)	0.25 ± 0.01^b^ (2.27%)	0.35 ± 0.03^b^ (1.46%)	0.18 ± 0.00^a^ (0.59%)
2,5-DHB	0.29 ± 0.02^b^ (3.72%)	0.02 ± 0.00^a^ (3.53%)	nd	0.54 ± 0.02^c^ (5.12%)
CA	0.24 ± 0.01^c^ (4.05%)	0.11 ± 0.00^a^ (0.51%)	0.12 ± 0.00^a^ (2.15%)	0.17 ± 0.01^b^ (1.06%)
3-HB	0.18 ± 0.01^b^ (4.81%)	0.05 ± 0.00^a^ (4.44%)	0.03 ± 0.00^a^ (3.01%)	0.17 ± 0.02^b^ (10.32%)
TPC	56.19 ± 1.28^a^ (2.27%)	24.09 ± 1.18^b^ (4.89%)	9.52 ± 0.99^c^ (10.30%)	10.64 ± 1.18^c^ (11.08%)

HH, heather honey; BH, buckwheat honey; RH, rapeseed honey; LH, linden honey; TPC, total polyphenol content; GA, gallic acid; 3,4-DHB, 3,4-dihydroxybenzoic acid; 2,5-DHB, 2,5-dihydroxybenzoic acid; CA, coumaric acid; 3-HB, 3-hydroxybenzoic acid

nd, not detected

### Evaluation of volatile compounds of nectar honeys

Table 3 shows the composition of the hypersurface phase of the honey samples. The following volatile compounds were identified: acetic acid ammonium acetate, isovaleraldehyde, hydroxyacetone, dimethylsilanediol, glyceraldehyde, furfural, arabinose, dihydroxyacetone, cyclopentane-1,2-dione, limonene, terpinene, sorbose, hydroxydihydromaltol, terpineol and levoglucosan (Figure S4). The common volatile compounds of all four honey types were acetic acid, glyceraldehyde and cyclopentane-1,2-dione. The highest number of volatile compounds was detected in buckwheat honey. Furthermore, buckwheat honey also contained the compounds belonging to terpenes, namely limonene, terpinene and terpineol which were not identified in other honey samples. Similarly, isovaleraldehyde, arabinose, levoglucosan were solely detected in buckwheat honey. Ammonium acetate was identified solely in rapeseed honey. Linden honey was the only one characterized by the content of hydroxyacetone, while heather honey contained hydroxydihydromaltol.

**Table 3:** Volatile compounds identified in the nectar honeys studied by GC-MS method

Number	Rt (min)	Compound	LH	BH	RH	HH
1	2.27	Acetic acid	x	x	x	x
2	2.34	Ammonium acetate			x	
3	2.90	Isovaleraldehyde		x		
4	3.08	Hydroxyacetone	x			
5	3.314	Dimethylsilanediol	x	x		
6	5.16	Glyceraldehyde	x	x	x	x
7	5.25	Furfural		x		x
8	5.98	Arabinose		x		
9	6.04	Dihydroxyacetone	x		x	x
10	6.47	Cyclopentane-1,2-dione	x	x	x	x
11	7.811	Limonene		x		
12	8.155	Terpinene		x		
13	8.98	Sorbose	x		x	x
14	9.09	Hydroxydihydromaltol				x
15	9.63	Terpineol		x		
16	12.28	Levoglucosan		x		

HH, heather honey; BH, buckwheat honey; RH, rapeseed honey; LH, linden honey

### TPC and antioxidant activity of honey samples

**Fig. 2:**
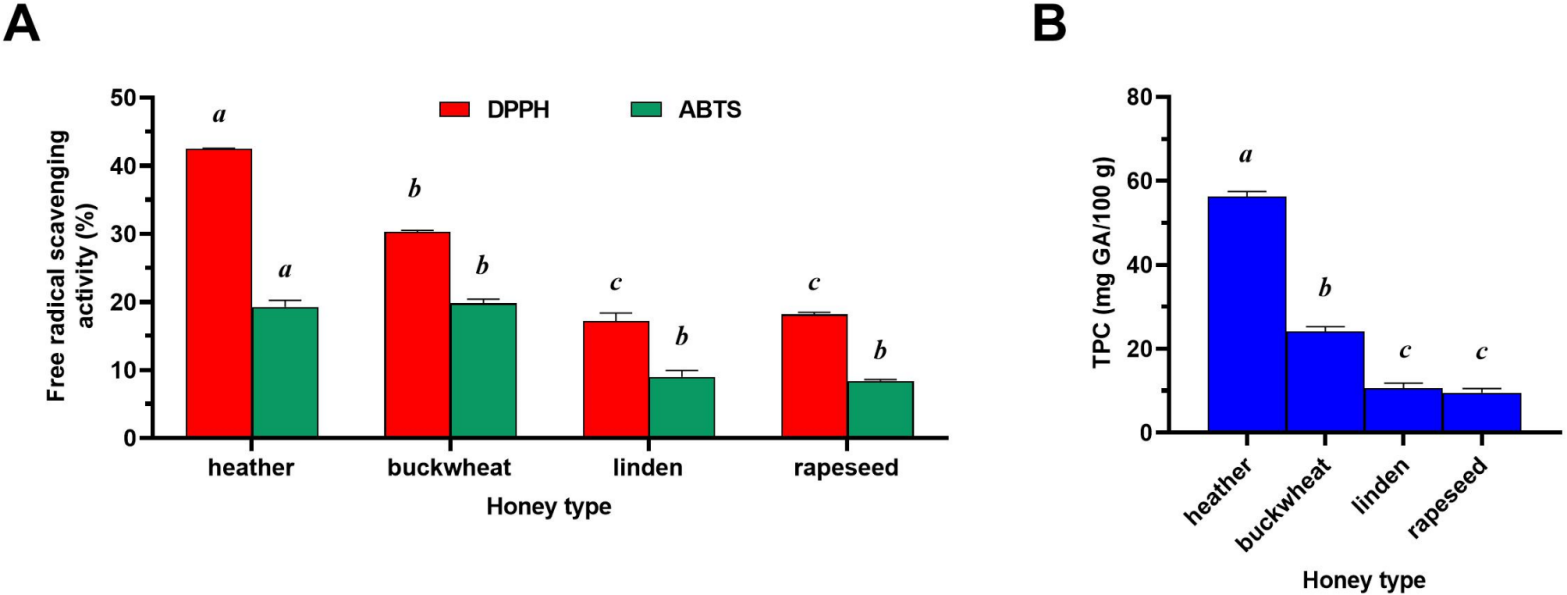
**(A)** The antioxidant activity of honey samples (*n* = 4) measured by the DPPH and ABTS assays and **(B)** total polyphenol content (TPC) of honey samples determined by the Folin–Ciocalteu method. Data are expressed as mean values with standard deviation. HH: heather honey; BH: buckwheat honey; LH: linden honey; RH: rapeseed honey. One-way ANOVA followed by Tukey’s post hoc test; different letters indicate significant differences between individual honeys, α = 0.05

The antioxidant activity of the analysed honey samples was determined using the DPPH and ABTS assays (Fig. 2A). All honey samples demonstrated free radical scavenging abilities. For the DPPH assay, the free radical scavenging activity ranged from 17.17% ± 1.18% for linden honey to 42.50% ± 0.06% for heather honey. For the ABTS assay, the scavenging activity ranged from 8.39% ± 0.20% for rapeseed honey to 19.79% ± 0.63% for buckwheat honey. In the case of DPPH method, heather honey exhibited significantly higher (*P*<0.001) antioxidant activity in compared to other honey samples. In the case of this honey, ​​were shown the highest RSA values, amounting to as much as 42.50 ± 0.06%. Buckwheat honey also showed high values (30.29 ± 0.21%). On the other hand, linden and rapeseed honeys showed significantly lower antioxidant activity with value of 17.17 ± 1.18% and 18.14 ± 0.32%, respectively. A similar trend was observed for the ABTS method, in which heather and buckwheat honey showed very similar RSA values, namely 19.24 ± 1.00% and 19.79 ± 0.63%, respectively. The antioxidant activity of these honeys was significantly higher in compared to linden and rapeseed honey, in which RSA value was very similar and amounted to 8.97 ± 0.96 and 8.39 ± 0.20% RSA (Fig. 2A). Figure 2B shows the TPC of the analysed honey samples, with values ranging from 9.52 ± 0.99 mg GA/100 g for rapeseed honey to 56.19 ± 1.28 mg GA/100 g for heather honey. Heather honey showed statistically significant the highest TPC in compared to other honeys. In this honey, the TPC value (56.19 ± 1.28 mg GA/100 g) was more than twice as high as in buckwheat honey (24.09 ± 1.18 mg GA/100 g) and five times as high as in linden honey (10.64 ± 1.18 mg GA/100 g) and rapeseed honey (9.52 ±. 0.99 mg GA/100 g) (Fig. 2B). Overall, heather honey exhibited significantly higher (*P* < 0.001) antioxidant activity and TPC than the other analysed honey samples.

Besides honey samples. the antioxidant activity of individual phenolic acids was also determined by DPPH and ABTS methods. Due to the fact that some of the standard substances showed very high antioxidant activity, the results were also expressed in mmol trolox/dm^3^. The results are shown in Table S1 and Figure S5.

A significant correlation was also found between DPPH and TPC (r_P_ = 0.9719) and between ABTS and TPC (r_P_ = 0.7699) (Figure S6).

### Cell toxicity of honey samples on skin fibroblasts

**Fig. 3:**
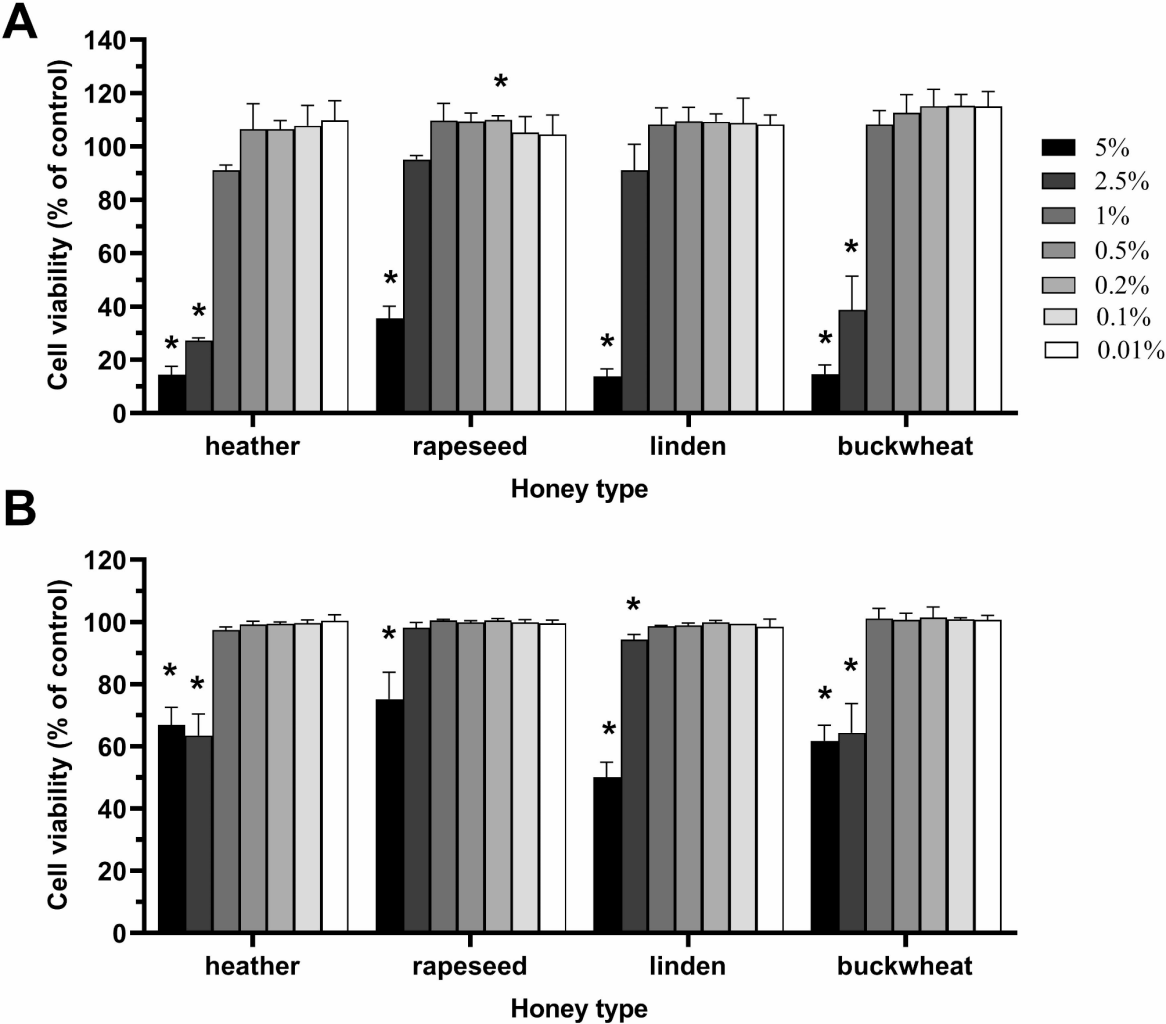
Effect of honey samples on mouse fibroblast L929 cell viability determined by **(A)** PrestoBlue assay and **(B)** LDH assay after 24 h of treatment. Data are expressed as mean values with standard deviation from three independent experiments. HH: heather honey; BH: buckwheat honey; LH: linden honey; RH: rapeseed honey. *P < 0.05 vs. control (Student’s t-test)

To evaluate the effect of honey samples on skin fibroblasts, a dose-response study was conducted using a murine fibroblast cell line (L929). Two assays, based on different mechanisms of action, were used to measure cell viability. As expected, both methods demonstrated a significant reduction in cell viability in compared to untreated control (*P* < 0.05) for all honey samples at the highest concentration tested (5%). Namely, heather, rapeseed, linden and buckwheat honey significantly decreased cell viability to 14.37 ± 3.12%, 35.53 ± 4.50%, 13.78 ± 2.73% and 14.60 ± 3.38%, respectively according to PrestoBlue assay as shown in Figs. 3A and 66.97 ± 5.62%, 75.16 ± 7.84%, 50.02 ± 5.14% and 61.81 ± 5.17%, respectively according to LDH assay, as shown in Fig. 3B. At subsequent tested concentrations, variations in the cytotoxicity of different honey types became apparent. Specifically, skin fibroblast viability was significantly reduced by heather and buckwheat honeys, as well as linden honey in the case of LDH assay, even at a concentration as low as 2.5%. Therefore, rapeseed honey was the most biocompatible, as confirmed by the highest IC_50_ value. According to the IC_50_ values, the toxicity of honey was as follows: 1.56 ± 0.14% < 2.00 ± 0.24% < 3.55 ± 0.61% < 3.78 ± 0.57% for heather, buckwheat, linden and rapeseed honey, respectively. These findings align with results from microscopy imaging. As shown in Figure S7, at a concentration of 5%, honey-treated cells appeared small and spherical, resembling cells treated with 0.9% lysis solution. In contrast, cells treated with rapeseed and linden honey at a concentration of 2.5%, and all honey samples at 1%, did not differ from untreated cells in terms of cell confluence and morphology.

### *In vitro* skin permeation and accumulation of honey phenolic acids

Skin permeation experiments were conducted using honey samples and honey-based hydrogels and emulsions. Initial stability and separation testing of both types of formulations demonstrated good physical properties. All prepared formulations were uniform in colour and showed no separation.

The results of skin permeation of phenolic acids from honey samples, honey-based hydrogels, and emulsions at 3, 5, and 24 h are presented in Table 4. As expected, the permeation capacity of phenolic acids increased with incubation time. Some phenolic acids were detectable in the acceptor fluid only after 5 h or even only after 24 h post-application. Additionally, some phenolic acids were not detected even after 24 h, such as 3-hydroxybenzoic acid in heather and rapeseed honey samples. Comparisons of honey and both honey-based formulations showed that hydrogel was by far the best phenolic acid releasing carrier.

**Table 4:** The content of phenolic acids in acceptor fluid after 3, 5 and 24 h penetration of heather honey and its hydrogel and emulsion formulations. The data are expressed as mean values ± SD and relative standard deviation (%RSD)

**Heather honey**						
**agent**	**time** **(h)**	**phenolic acid** **µg/cm** ^**2**^ **(%RSD)**				
		**GA**	**3,4-DHB**	**2,5-DHB**	**CA**	**3-HB**
**Heather** **honey**	3	0.70 ± 0.09 (12.95%)	nd	nd	nd	nd
	5	3.17 ± 0.48 (15.00%)	1.04 ± 0.31 (29.45%)	1.21 ± 0.12 (9.56%)	0.32 ± 0.11 (33.26%)	nd
	24	15.62 ± 1.43 (9.15%)	2.18 ± 0.98 (45.07%)	2.64 ± 0.22 (8.51%)	1.42 ± 0.14 (10.10%)	nd
**Hydrogel**	3	3.12 ± 0.78 (25.05%)	1.55 ± 0.24 (15.79%)	nd	nd	0.99 ± 0.11 (11.42%)
	5	8.66 ± 2.22 (25.58%)	2.57 ± 0.36 (14.16%)	1.17 ± 0.14 (12.43%)	nd	1.56 ± 0.28 (17.68%)
	24	21.72 ± 1.52 (6.99%)	5.14 ± 1.28 (24.88%)	4.02 ± 0.93 (23.26%)	1.64 ± 0.01 (0.44%)	3.69 ± 0.39 (10.51%)
**Emulsion**	3	nd	nd	nd	nd	nd
	5	1.93 ± 0.13 (6.50%)	0.87 ± 0.29 (33.65%)	nd	nd	1.37 ± 0.05 (3.64%)
	24	10.73 ± 1.80 (16.78%)	3.56 ± 0.23 (6.40%)	1.67 ± 0.36 (21.62%)	1.18 ± 0.12 (10.09%)	3.58 ± 0.03 (0.80%)
**Buckwheat honey**						
**agent**	**time** **(h)**	**phenolic acid** **µg/cm** ^**2**^ **(%RSD)**				
		**GA**	**3,4-DHB**	**2,5-DHB**	**CA**	**3-HB**
**Buckwheat** **honey**	3	nd	nd	nd	nd	nd
	5	1.38 ± 0.16 (11.70%)	nd	1.51 ± 0.14 (9.56%)	nd	nd
	24	11.52 ± 2.31 (20.05%)	2.92 ± 0.21 (7.20%)	3.67 ± 0.40 (10.92%)	0.55 ± 0.07 (13.48%)	nd
**Hydrogel**	3	1.32 ± 0.22 (16.93%)	nd	nd	nd	nd
	5	2.27 ± 0.19 (8.58%)	1.01 ± 0.25 (24.42%)	nd	nd	nd
	24	14.80 ± 1.61 (10.92%)	11.62 ± 1.33 (11.47%)	nd	nd	1.85 ± 0.21 (11.59%)
**Emulsion**	3	nd	nd	0.91 ± 0.22 (23.89%)	nd	nd
	5	0.76 ± 0.07 (9.40%)	nd	1.51 ± 0.14 (9.56%)	nd	nd
	24	6.42 ± 0.21 (3.33%)	1.71 ± 0.20 (11.49%)	3.67 ± 0.40 (10.92%)	0.47 ± 0.11 (24.05%)	2.26 ± 0.32 (14.07%)
**Rapeseed honey**						
**agent**	**time** **(h)**	**phenolic acid** **µg/cm** ^**2**^ **(%RSD)**				
		**GA**	**3,4-DHB**	**2,5-DHB**	**CA**	**3-HB**
**Rapeseed** **honey**	3	nd	nd	nd	nd	nd
	5	1.35 ± 0.25 (18.42%)	1.31 ± 0.38 (29.45%)	nd	nd	nd
	24	11.52 ± 2.31 (19.83%)	4.52 ± 0.35 (7.71%)	nd	0.47 ± 0.15 (33.19%)	nd
**Hydrogel**	3	nd	nd	0.91 ± 0.22 (23.89%)	nd	nd
	5	4.792 ± 1.33 (27.70%)	1.31 ± 0.38 (29.45%)	1.51 ± 0.14 (9.56%)	nd	nd
	24	22.21 ± 1.73 (7.80%)	4.52 ± 0.35 (7.71%)	3.67 ± 0.40 (10.92%)	0.47 ± 0.11 (24.05%)	2.26 ± 0.32 (14.07%)
**Emulsion**	3	nd	nd	nd	nd	nd
	5	0.71 ± 0.05 (7.31%)	1.31 ± 0.38 (29.45%)	nd	nd	nd
	24	11.56 ± 1.87 (16.15%)	6.00 ± 0.90 (14.95%)	nd	nd	2.21 ± 0.26 (11.74%)
**Linden honey**						
**agent**	**time** **(h)**	**phenolic acid** **µg/cm** ^**2**^ **(%RSD)**				
		**GA**	**3,4-DHB**	**2,5-DHB**	**CA**	**3-HB**
**Linden** **honey**	3	nd	nd	nd	nd	nd
	5	1.23 ± 0.27 (21.63%)	0.97 ± 0.21 (21.58%)	1.27 ± 0.24 (19.27%)	nd	1.04 ± 0.21 (21.21%)
	24	14.53 ± 1.48 (10.16%)	3.40 ± 0.21 (6.18%)	3.69 ± 0.39 (10.50%)	0.70 ± 0.10 (14.33%)	3.77 ± 0.24 (6.39%)
**Hydrogel**	3	nd	nd	nd	nd	nd
	5	1.547 ± 0.10 (6.53%)	1.04 ± 0.31 (29.45%)	0.47 ± 0.10 (21.27%)	nd	nd
	24	8.587 ± 1.44 (16.78%)	10.00 ± 2.28 (22.77%)	1.72 ± 0.09 (4.97%)	0.38 ± 0.09 (24.05%)	1.90 ± 0.34 (17.79%)
**Emulsion**	3	0.38 ± 0.10 (27.71%)	nd	nd	nd	nd
	5	0.58 ± 0.01 (1.48%)	nd	1.21 ± 0.12	nd	nd
	24	1.40 ± 0.12 (8.43%)	1.40 ± 0.23 (16.72%)	2.94 ± 0.32 (10.92%)	nd	2.12 ± 0.36 (18.88%)

GA, gallic acid; 3,4-DHB, 3,4-dihydroxybenzoic acid; 2,5-DHB, 2,5-dihydroxybenzoic acid; CA, coumaric acid; 3-HB, 3-HB; nd, not detected

**Fig. 4:**
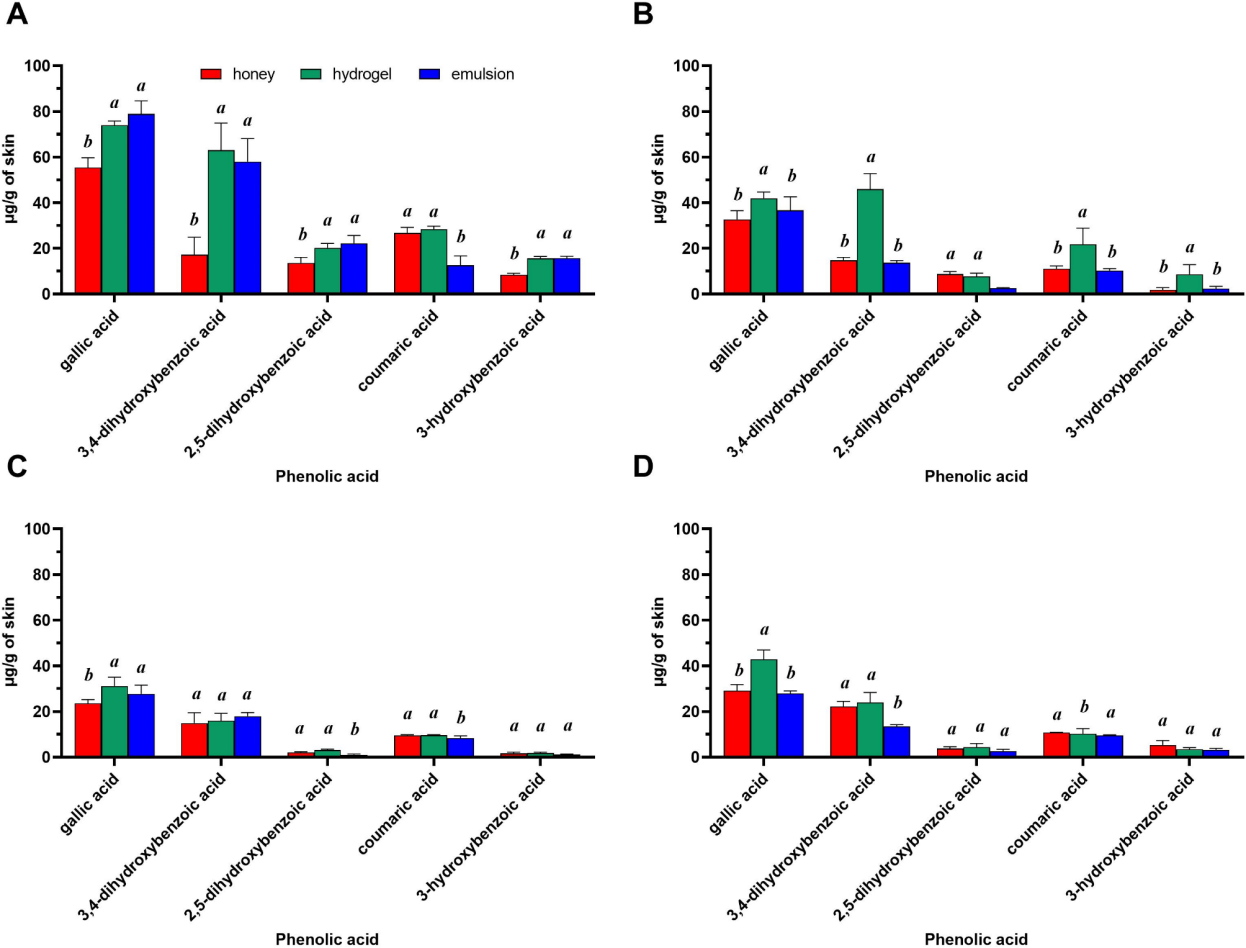
Accumulation of phenolic acids in the skin after 24-hour penetration studies. **(A)** Heather honey, **(B)** Buckwheat honey, **(C)** Linden honey, **(D)** Rapeseed honey. Data are expressed as mean values with standard deviation from three independent experiments. One-way ANOVA followed by Tukey’s post hoc test; different letters indicate significant differences between individual forms applied to the skin, α = 0.05

In the case of heather honey, the GA content in the acceptor fluid collected after 24 h of penetration was 21.72 ± 1.52 µg/cm^2^ and was higher in compared to the emulsion (10.73 ± 1.80 µg/cm^2^) and pure honey (15.62 ± 1.43 µg/cm^2^). After application the hydrogel containing heather honey after 24 h of testing, GA permeated the skin in quantity of 7.77%, then 3.84% from the emulsion and 0.70% for pure honey. A similar trend was observed in the case of other honeys, where permeation of GA after using the hydrogel with buckwheat honey was 14.80 ± 1.61 µg/cm^2^, which was 15.58% in compared to the emulsion (6.75%) and pure honey (1.21%). However, after applying the hydrogel with rapeseed honey to the skin, GA permeated in the amount of 22.21 ± 1.73 µg/cm^2^ (22.61%), which was almost twice as high as compared to the emulsion (11.77%) and almost twenty times higher in compared to pure honey (2.26%). In the case of the hydrogel containing honey linden honey, GA penetration was 31.85; 5.20 and 4.31% for the hydrogel, emulsion and pure honey, respectively. It was also observed the high 3,4-DHB penetration from the hydrogel containing heather honey (14.90%), slightly lower from the emulsion (10.32%) and almost two times lower from pure honey (7.882%). The high penetration of 3,4-DHB was also observed from the hydrogel containing heather honey (14.90%), slightly lower from the emulsion (10.32%) and almost twice lower from pure honey (7.88%). Whereas in the case of the hydrogel containing buckwheat honey, the penetration of 3.4DHB was almost twenty times higher (20.57%) compared to pure honey (1.29%) (Table 4). The accumulation of individual phenolic acids in the skin after 24 h of permeation is shown in Fig. 4. Following the application of all formulations, accumulation of phenolic acids in the skin was observed; however, the extent varied depending on the vehicle used. The highest accumulation of GA in the skin was found after the application of the emulsion containing heather honey, with a GA content of 78.85 ± 5.82 µg/g of skin. Additionally, a high accumulation of GA (73.98 ± 1.87 µg/g of skin) was noted after the application of the hydrogel containing heather honey. The second most accumulated phenolic acid was 3,4-DHB, with the greatest accumulation in most cases observed following the application of honey-based hydrogels (Fig. 4).

### Biodegradation of honey-based formulations

Table 5 presents the results of the biodegradation tests for the formulations using bacterial cultures. After 28 days, the highest percentage of biodegradation was observed in the reference sample SDS, with 91 ± 3%, classifying it as category 01 (easily biodegradable) according to OECD standards. Among the honey-based formulations, biodegradation percentages were as follows: 32% ± 0% for emulsion with linden honey and emulsion with rapeseed honey, 31% ± 1% for emulsion with heather honey, 30% ± 2% for emulsion with rapeseed honey, 29% ± 1% for hydrogel with linden honey and hydrogel with rapeseed honey, 28% ± 1% for hydrogel with heather honey, and 26% ± 1% for hydrogel with buckwheat honey. These results classify all honey-based formulations into category 02 (partially biodegradable) per OECD guidelines.

**Table 5:** Biodegradation of honey-based hydrogels and emulsion formulations by bacterial cultures. The data are expressed as mean values with SD in percentage of biodegradability. Three independent experiments were performed, during each of which the collected samples were analyzed three times

Time (day)	Biodegradation degree (%)								
	Honey-based formulations								SDS
	HHH	HLH	HBH	HRH	EHH	ELH	EBH	ERH	
0	0 ± 0	0 ± 0	0 ± 0	0 ± 0	0 ± 0	0 ± 0	0 ± 0	0 ± 0	0 ± 0
1	1 ± 0	3 ± 0	1 ± 0	3 ± 0	3 ± 0	2 ± 0	2 ± 0	2 ± 0	10 ± 0
3	3 ± 0	6 ± 1	3 ± 0	6 ± 1	16 ± 2	10 ± 1	5 ± 1	10 ± 2	26 ± 1
6	10 ± 1	11 ± 2	9 ± 1	9 ± 1	15 ± 2	16 ± 2	10 ± 0	16 ± 2	49 ± 2
8	13 ± 2	15 ± 1	11 ± 1	16 ± 2	15 ± 0	17 ± 1	13 ± 2	17 ± 0	49 ± 1
11	19 ± 2	22 ± 3	18 ± 1	22 ± 2	24 ± 2	25 ± 0	20 ± 0	25 ± 2	51 ± 4
14	21 ± 3	25 ± 2	20 ± 2	25 ± 2	29 ± 0	29 ± 2	24 ± 3	29 ± 4	68 ± 1
17	22 ± 1	28 ± 1	20 ± 2	28 ± 3	29 ± 3	30 ± 3	25 ± 0	30 ± 0	74 ± 3
19	28 ± 2	32 ± 2	25 ± 2	32 ± 2	30 ± 0	30 ± 0	25 ± 0	30 ± 3	76 ± 3
21	27 ± 1	29 ± 3	24 ± 3	32 ± 1	30 ± 3	31 ± 2	25 ± 0	30 ± 0	82 ± 4
24	27 ± 2	29 ± 0	24 ± 1	33 ± 0	29 ± 3	30 ± 0	26 ± 0	30 ± 3	92 ± 1
27	27 ± 0	29 ± 1	24 ± 2	29 ± 0	31 ± 0	32 ± 3	32 ± 0	32 ± 2	91 ± 2
28	28 ± 1	29 ± 1	26 ± 1	29 ± 1	31 ± 1	32 ± 2	30 ± 2	32 ± 2	91 ± 3
Class*	02	02	02	02	02	02	02	02	01

*Biodegradability class in the aquatic environment Categories: „01” - easily biodegradable, biodegradation rate after 10 days ≥ 60% in a 28-day test; „02” - partially biodegradable, biodegradation rate greater than 20% but less than 60% in a 28-day test

HHH, hydrogel with heather honey; HBH, hydrogel with buckwheat honey; HLH, hydrogel with linden honey; HRH, hydrogel with rapeseed honey; EHH, emulsion with heather honey; EBH, emulsion with buckwheat honey; ELH, emulsion with linden honey; ERH, emulsion with rapeseed honey; SDS, sodium dodecyl sulphate

## Discussion

Honey polyphenols, in particular, have attracted considerable attention in wound treatment because of their antimicrobial, regenerative, and antioxidant properties [13]. These properties of honey, as well as its valuable polyphenol composition, are reflected in their effective use in the cosmetics industry. However, their effectiveness depends on their ability to permeate the skin layers and accumulate within the skin [40].

In present study, we initially analysed the composition of phenolic acids using the HPLC method and volatile compounds using the GC-MS method in four honey samples. Five phenolic acids were identified, namely GA, 3,4-DHB, 2,5-DHB, CA and 3-HB. The highest content of phenolic acids was found in heather honey. In previous studies, 3-HB, 3,4-DHB and CA as well as CA derivatives were determined in Polish buckwheat and heather honeys [41–43]. Similarly, GA, 3,4-DHB and CA were identified in heather honey from Turkey [44].

Volatile compounds found in honey are aromatic substances obtained from bees-collected nectar. In our study, the common volatile compounds of all honey samples were acetic acid, glyceraldehyde and cyclopentane-1,2-dione. Acetic acid has recently been identified in honeybee products derived from medicinal plants such as marjoram and citrus [45]. Ammonium acetate was identified only in rapeseed honey, while hydroxyacetone was found only in linden honey. This unique volatile compound was identified using headspace GC-MS in a honeybee product from marjoram, while hydroxyacetone was identified in the product Citrus royal jelly [45].

Some varieties of nectar of manuka tree (*Leptospermum scoparium*), i.e. single-flowered red or pink flowers, produce a very high levels of dihydroxyacetone in the nectar [46]. Our research showed that dihydroxyacetone is also present in linden, rapeseed and heather honey. It has been shown elsewhere [45] that some compounds such as furfural appeared in marjoram, trifolium and citrus honey, while in present study furfural was identified only in buckwheat and heather honey. Two monoterpenes (terpinene and limonene) are among the most abundant volatile compounds in the honeybee bread samples, which have not been previously described, but the monoterpenoid terpineol is also present [47]. Moreover, limonene was also present in honeybee products from marjoram and trifolium plants and in our buckwheat honey. In this honey type, monosaccharide arabinose was also identified, whereas a monosaccharide sorbose was found in the other three types of honey tested.

Antioxidant activity is a crucial property for newly developed skin formulations [29]. The skin is constantly exposed to oxidative stress from reactive oxygen species, which damage cellular components such as DNA, lipids, and proteins. Previous studies have shown that heather honey from Poland also exhibits the highest TPC [48, 49] and antioxidant activity [48]. However, heather honey samples from different regions of Poland had lower TPC and antioxidant activity than honeydew and buckwheat honeys [41], likely due to differences in geographical location and soil type [50].

In our study, all honey samples showed the ability to scavenge free radicals. Heather honey showed the highest antioxidant activity, both in the DPPH and the ABTS method. A similar trend was observed in the case of TPC, in which the TPC in heather honey was almost six times higher in compared to rapeseed honey. The antioxidant activity of popular Polish honeys, including polyfloral, rapeseed, buckwheat, acacia and honeydew honey was also confirmed in other studies [51, 52]. In these studies, buckwheat honey showed the highest antioxidant activity, TPC and the highest content of individual phenolic acids such as p-hydroxybenzoic acid, caffeic acid, CA, vanillic acid. In our study, the highest antioxidant activity of heather honey was probably also caused by, among others, the higher content of some phenolic acids, such as GA and 3-HB that are strong natural antioxidants [53, 54] which was also confirmed by our analysis of antioxidant activity of pure standards. In our study, a significant correlation was shown between the methods of antioxidant activity and TPC indicating that the total pool of polyphenols is responsible for scavenging free radicals. However, when analysing the total content of phenolic acids, it was observed that they represent only a minor part of TPC. For example, in heather honey, the content of determined phenolic acids was only 6.87% in relation to TPC. Similar results were published in other studies, where the overall content of the seven determined phenolic acids (among others 4-HB, 3,4-DHB and CA) in buckwheat honey was only 1.63% in relation to the TPC; while in heather honey it was 3.42% in relation to the TPC of this honey [41]. In other studies, the total content of six phenolic acids (among others GA and 3,4-DHB) and one flavonoid in Turkish heather honey showed only 8.84% of the TPC [44]. Similar proportions are also confirmed by study analyzing the composition of 14 Polish heather honeys [43]. In two selected honeys, the marked phenolic acids (among others 3-HB, 3,4-DHB, CA and FA) constituted only 7.84% and 6.62% in relation to the TPC content. Similar results were also observed in other 12 honey samples.

The major phenolic acid of heather honey that appeared in heather honey samples of different geographical origin is an isoprenoid, abscisic acid [43]. Its content was very high and represents about 44.27–68.92% of the total phenolic acids content [43, 55]. When analysing the composition of heather honey in our study, we observed a very high and sharp peak at 6.34 min on the HPLC-UV chromatogram and most likely represents abscisic acid (Figure S4). However, due to the lack of access to the abscisic acid standard, we could not confirm it quantitatively. The large difference between the content of total phenolic acids and TPC may be due to the content of other honey components that react with the Folin-Ciocalteu reagent, such as compounds belonging to terpenes including abscisic acid. Furthermore, the Folin-Ciocalteu reagent can react not only with phenols, but also with other reducing compounds such as amino acids, sugars and vitamin C [56, 57].

Among honey phenolic compounds, flavonoids and phenolic acids (including benzoic and cinnamic acid derivatives) are the most significant polyphenols, exhibiting various biological activities [58]. Some of them, such as GA, CA or chlorogenic acid, occur in various honeys regardless of their origin [59–62]. In this study, GA was the dominant phenolic acid in the analysed honey samples, with the highest concentration found in heather honey. GA was also the most highly accumulated phenolic acid in the skin, displaying potent antioxidant, anti-inflammatory, antimicrobial, and anticancer activities [17], and it has been extensively studied for its role in dermatological conditions and wound healing. Strong evidence shows that GA accelerates wound healing *in vitro* and *in vivo* [19–21] and inhibits proteolytic enzymes such as elastases and collagenases, which impair wound healing [63]. Moreover, this compound can inhibit melanin production and thus it is an effective topical depigmenting or skin lightening cosmetic [22]. Other phenolic acids identified in the honeys analyzed may also have beneficial effects on the skin. For example, 3,4-DHB may protect skin cells from damage caused by ultraviolet B (UVB) radiation [64] and may exhibit anti-wrinkle activity by inducing type I collagen synthesis in human dermal fibroblasts and skin explants [24]. CA may also have hyperpigmentation effects and may have applications in skin lightening formulations by inhibiting cell melanogenesis [65].

Experiments using porcine skin in Franz diffusion cells showed that GA and its derivatives penetrate all skin layers, especially the SC, where they exert antioxidant effects [66], which is particularly important when using anti-aging preparations. Reactive oxygen species (ROS) are one of the main factors contributing to skin aging. Therefore, natural antioxidants provided in preparations applied to the skin play an important role, as they can limit the negative effects of ROS [67].

However, as demonstrated here and elsewhere [28], the permeation capacity of GA and other phenolic acids is affected by several factors. The biological activity of formulations applied to the skin primarily depends on the permeation capacity of their active ingredients. Effective topical penetration requires the release of these substances from the formulation to reach all skin layers [68]. In this study, the penetration and accumulation of phenolic acids varied based on the type of honey and the vehicle used. Pure honeys and two types of pharmaceutical formulations containing 10% honey were tested. Literature indicates that honey is commonly used in concentrations ranging from 1 to 10% in products such as lip ointments, creams, gels, lotions, shampoos, and conditioners [69]. The highest skin penetration capacity was generally observed in honey-based hydrogels. This finding suggests that the formulation type plays a crucial role in skin application. Other studies regarding permeation of propolis phenolic acids in three different vehicles showed that the hydrogel released almost the entire content of phenolic compounds after 8 h, while only up to 5% and 22% of phenolic compounds were released from the ointments and the w/o emulsion. Greater penetration from the hydrogel matrix can be associated, among other factors, with the lipophilicity of the semisolid carrier, which dissolves better after penetration into the aqueous acceptor medium [70]. In addition, hydrogels applied to the skin can retain a significant amount of water that plays an important role in skin hydration. The moisturizing the SC of the epidermis facilitates the delivery of the active ingredient deep into the skin [71]. In our study, an additional factor that could have influenced greater penetration with hydrogel was the glycerine used in its composition. Glycerine is often considered to be a promoter of absorption of active substances through the skin [72]. Glycerine is known to penetrate the SC and retain water in the skin. After penetration into the skin forms a “reservoir” deep in the SC in lipid bilayers without disrupting its structure. This is called “swelling,” characterized by intracellular expansion of corneocytes and intercellular expansion between corneocytes. This mechanism probably results in improved skin barrier properties as well as water retention capacity in the skin, which results in a moisturizing effect [73]. Therefore, glycerine increases the bioavailability of phenolic acids by affecting membrane permeability [74]. Considering the penetration ability of active substances in present study, the emulsion proved to be less effective vehicle. Nevertheless, phenolic acids penetrated better from the emulsion than from pure honey. In this case, grape seed oil, a primary ingredient in the prepared emulsions, may further increase the penetration due to its fatty acids, which enhance skin permeability by liquefying the SC [75]. In this study, phenolic acids in pure honey penetrated the skin at levels lowers to formulations containing 10% honey. The SC, rich in fatty components, poses a barrier to hydrophilic substances such as honey, complicating their penetration. To date, no study has characterised the permeation profile of honey polyphenols. Propolis, another bee product rich in polyphenols, has been studied for phenolic compound penetration into the skin [70, 76–78], with techniques such as micro- and nano-emulsions developed to enhance intradermal penetration [70, 77]. Different penetration profiles of phenolic acids were observed in our study depending on the type of honey. In the case of the hydrogel with heather honey, 7.77% of GA permeated, while in the case of the hydrogel with buckwheat honey 15.85% was found to be permeated, while in the case of rapeseed honey it is as much as 22.21%. Similarly, 3,4-DHB penetrated at an amount of 20.57% from the hydrogel containing buckwheat honey, while 15.58% from the hydrogel containing heather honey. Even though the vehicle was the same for both honey types, the permeation of GA and 3,4-DHB were different. Different composition of both honey types could be a responsible factor affecting the different penetration. High variety and diverse concentrations of secondary metabolites in honey samples of different botanical origin can either increase or decrease the permeation capacity. The composition of heather honey is very characteristic and the main component of heather honey that distinguishes it from other honey types is abscisic acid and its derivatives [55]. In addition, honeys of various botanical origins may contain different amounts of other ingredients, such as sugars, enzymes, amino acids, organic acids, volatile compounds or free fatty acids. In our study, GC-MS analysis revealed the content of three terpenes (limonene, terpinene and terpineol) in buckwheat honey that may be responsible for the greater penetration of GA and 3,4-DHB from the hydrogel containing this honey type. Terpenes found in essential oils can increase the penetration of active substances through the skin, primarily by interacting with SC lipids [79]. Other factors affecting greater penetration may include different physicochemical properties of individual honey type. In addition, the lipophilicity of a given phenolic acids and the formulation used also play a major role. Phenolic acids are partially lipophilic by nature and therefore they can penetrate differently depending on the environment [70]. There are not many reports in the available literature on the skin penetration of phenolic acids contained in various types of honey. The permeation of phenolic acids was analysed from propolis contained in three pharmaceutical formulations, such as ointment and w/o emulsion, with varying degrees of permeation observed depending on the formulation. The authors suggested that the main role in the penetration of phenolic acids is their lipophilicity and therefore more hydrophilic compounds are released better, especially from formulations containing no lipophilic substances. For example, CA was released and penetrated into the skin in lower amounts compared to vanillic acid which has lower lipophilicity [70]. This is confirmed by our studies, in which GA and 3,4-DHB characterized by low lipophilicity (GA - log P 0.7; 3,4-DHB - log P 0.86) penetrated most often in the greatest amount compared to CA which is characterized by higher lipophilicity (log *P* − 1.79). In contrast, Hossain et al. [80] observed the comparable released profiles of active components from five different floral honey types and their formulations (alginate-based honey formulations). Similarly, the release of methylglyoxal, a major active compound in manuka honey, has been evaluated in various manuka honey samples and their formulations [81]. In this study, over 90% of the initial methylglyoxal content was released from the most manuka honey samples within 12 h, with approximately 30% released within the first hour of application. The authors used a dialysis membrane, which is why they obtained such a high percentage of active substance release from honey. In the case of analysis of penetration through human or porcine skin, penetration into the acceptor fluid would probably be lower.

Topical honey is considered safe with low toxicity [69]. *In vitro* studies have shown minimal cytotoxicity on skin cells, including keratinocytes and fibroblasts [82]. At concentrations of 1% for heather, linden, and buckwheat honeys and 2.5% for rapeseed honey, cell viability and morphology did not differ from untreated cells. In contrast, Samarghandian et al. [83] reported no decrease in cell viability even at 15% honey concentration after 24 h, with discrepancies likely due to different honey types and concentrations. Despite longer incubation (48 h), Sadeghi‑Aliabadi et al. [84] reported similar IC_50_ values for Astragalus honey compared with the Polish honeys analysed herein. According to the results of the PrestoBlue^®^ assay, cell viability ranged from 14.4% ± 3.1% for heather honey to 35.5% ± 4.5% for rapeseed honey at the highest tested concentration (5%). In contrast, the LDH assay showed cell viability ranging from 49.8% ± 4.9% for 5% linden honey to 75.8% ± 8.7% for rapeseed honey at the same concentration. These discrepancies suggest a higher proportion of metabolically inactive cells compared to dead cells with damaged membranes after 24 h of treatment when compared to untreated cells. It is presumed that extending the incubation time with the highest tested doses would increase the toxicity observed during the LDH assay. Additionally, in the LDH assay, the differences in cell viability within the tested concentration range were insufficient to determine the IC_50_. Regardless of the method used to determine cell viability, rapeseed honey was completely safe at concentration of 2.5%, whereas other honeys at concentration of 1%.

In this study, the prepared honey-based hydrogels and emulsions were also tested for their biodegradability, aligning with the European Green Deal’s emphasis on sustainable impact, especially concerning cosmetics. Our findings showed that the analysed formulations were partially biodegradable, marking an important step toward designing sustainable cosmetic products that fulfil their intended function while minimising environmental impact, particularly regarding environmental protection as part of sustainable development’s third pillar [85].

## Conclusions

In summary, our findings confirmed that Polish honey samples, which exhibit high antioxidant activity and a high content of phenolic acids, possess beneficial skin properties. Among the tested honeys, heather honey had the highest content of phenolic acids, particularly GA. Honey phenolic acids were easily released from the prepared honey-based hydrogels and emulsions and after their initial accumulation in the skin, they penetrated deeper skin layers. Hydrogel was shown to be the most suitable vehicle for releasing honey phenolic acids and most interestingly, permeation through the skin from this vehicle was even several times higher in compared to honey pure.

In this study, the highest permeation through the skin was mostly observed with hydrogels containing rapeseed honey. Interestingly, a high concentration of phenolic acids in heather honey did not reflect a greater penetration of these compounds through the skin, especially in the case of GA. Therefore, further studies are needed to clarify the variation in permeation of phenolic acids from different honey types. Furthermore, other phytochemicals such as sugars, fatty acids, amino acids and volatile compounds may increase or decrease the permeation of phenolic acids. Therefore, detailed chemical analysis of honeys is required in future research, allowing an accurate explanation of the differences in the penetration of active substances depending on the type of honey. Nevertheless, the hydrogels used in the study proved to be the most beneficial formulation. Since hydrogels are becoming increasingly popular in anti-wrinkle, anti-inflammatory or antioxidant preparations, in combination with honey they can be an innovative solution in delivering phenolic compounds to the skin. Due to the hydrophilic nature of this formulation, it can have a simple composition and be easy to use. In present study, honeys, at the highest concentration used (5%), did not exhibit toxicity to human fibroblasts. Finally, prepared honey-based formulations were partially biodegradable. This study serves as a starting point for further research aimed at developing natural cosmetic and dermatological preparations containing honey. Although our study is innovative, but it has certain limitations that should be considered in future analyses. First, it lacks a very detailed analysis (e.g. LC-UV-MS), which would allow us to determine a wider range of active ingredients in honeys. Another limitation of this study is the design of the preparations containing honey with different botanical origin and thus with different chemical composition. Therefore, it is necessary to conduct a thorough phytochemical analysis of honeys applied to the skin in future study and then a detailed interpretation of which of the ingredients contained could have contributed to the possible beneficial effects on the skin.

## Electronic supplementary material

Below is the link to the electronic supplementary material.

Supplementary Material 1

Supplementary Material 2

Supplementary Material 3

Supplementary Material 4

Supplementary Material 5

Supplementary Material 6

Supplementary Material 7

Supplementary Material 8

## Data Availability

The datasets used and/or analysed during the current study are available from the corresponding author on reasonable request.

## Abbreviations

3,4-DHB: 3,4-dihydroxybenzoic acid
2,5-DHB: 2,5-dihydroxybenzoic acid
3-HB: 3-hydroxybenzoic acid
ABTS: 2,2′-azino-bis(3-ethylbenzothiazoline-6-sulfonic) acid
BH: Buckwheat honey
CA: Coumaric acid
DPPH: 2,2-Diphenyl-1-picrylhydrazyl
EBH: Emulsion with buckwheat honey
EHH: Emulsion with heather honey
ELH: Emulsion with linden honey
ERH: Emulsion with rapeseed honey
FA: Ferulic acid
GA: Gallic acid
HBH: Hydrogel with buckwheat honey
HH: Heather honey
HHH: Hydrogel with heather honey
HLH: Hydrogel with linden honey
HPLC: High-performance liquid chromatography
HRH: Hydrogel with rapeseed honey
LH: Linden honey
LDH: Lactate dehydrogenase
RH: Rapeseed honey
ROS: Reactive oxygen species
SC: Stratum corneum
SDS: Sodium dodecyl sulphate
TPC: Total polyphenol content
