# Supplementary material for: Assessment of in vitro skin permeation and accumulation of phenolic acids from honey and honey-based pharmaceutical formulations
Source: BMC Complement Med Ther. 2025 Feb 4;25:43. doi: 10.1186/s12906-025-04786-1 (PMC11796271; doi:10.1186/s12906-025-04786-1)
Supplement: Supplementary file 1 — Supplementary Material 1 [file 12906_2025_4786_MOESM1_ESM.docx]

**Table S1.** Antioxidant activity of pure patterns used for identification of phenolic acids in honeys. The data are expressed as the mean values with SD (n = 3).

| **Standards** | **DPPH** | | **ABTS** | |
| --- | --- | --- | --- | --- |
|  | **(mmol/dm^3^)** | **(% RSA)** | **(mmol/dm3)** | **(% RSA)** |
| gallic acid | 11.27 ± 0.01 | 67.73 ± 0.76* | 21.47 ± 0.59 | 29.86 ± 0.80* |
| 3,4-dihydroxybenzoic acid | 9.61 ± 0.01 | 59.00 ± 1.51* | 7.73 ± 0.21 | 11.18 ± 0.29* |
| 2,5-dihydroxybenzoic acid | 9.36 ± 0.00 | 57.12 ± 0.91* | 14.08 ± 1.22 | 19.82 ± 1.65* |
| coumaric acid | 0.24 ± 0.01 | 27.24 ± 1.09 | 1.75 ± 0.03 | 48.28 ± 0.86 |
| 3-hydroxybenzoic acid | 0.16 ± 0.00 | 19.18 ± 0.82 | 0.49 ± 0.04 | 14.14 ± 1.04 |

The standards for analysis were used at concentration of 0.005 % and dissolved in 70% ethanol (v/v).

* stock samples of compounds (0.005% concentration) gallic acid, 3,4-dihydroxybenzoic acid and 2,5-dihydroxybenzoic acid were diluted 20-fold. The reason for the dilution was the very high activity of the compounds, which after 20-fold dilution still brightened the DPPH reagent.
