# Supplementary figures and images for: Assessment of in vitro skin permeation and accumulation of phenolic acids from honey and honey-based pharmaceutical formulations
Source: BMC Complement Med Ther. 2025 Feb 4;25:43. doi: 10.1186/s12906-025-04786-1 (PMC11796271; doi:10.1186/s12906-025-04786-1)

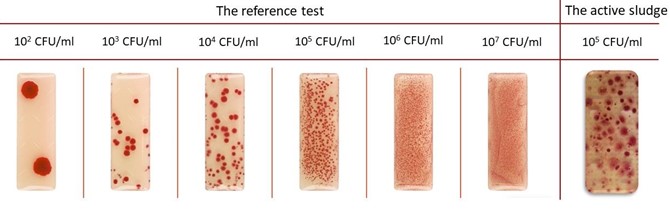

Supplement: Supplementary file 2 — Supplementary Material 2 [file 12906_2025_4786_MOESM2_ESM.jpg]

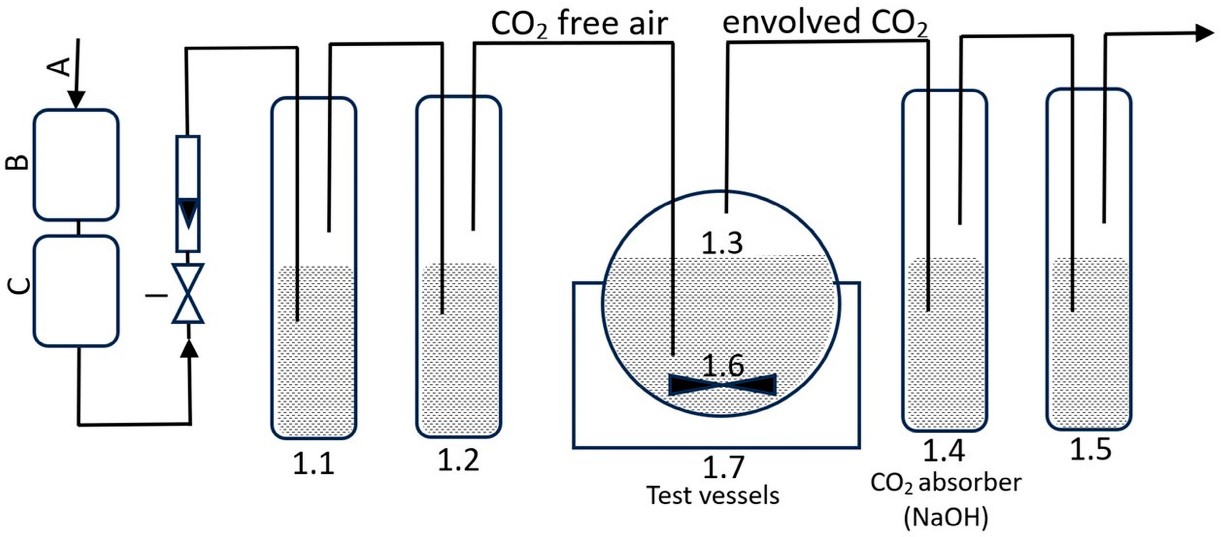

Supplement: Supplementary file 3 — Supplementary Material 3 [file 12906_2025_4786_MOESM3_ESM.jpg]

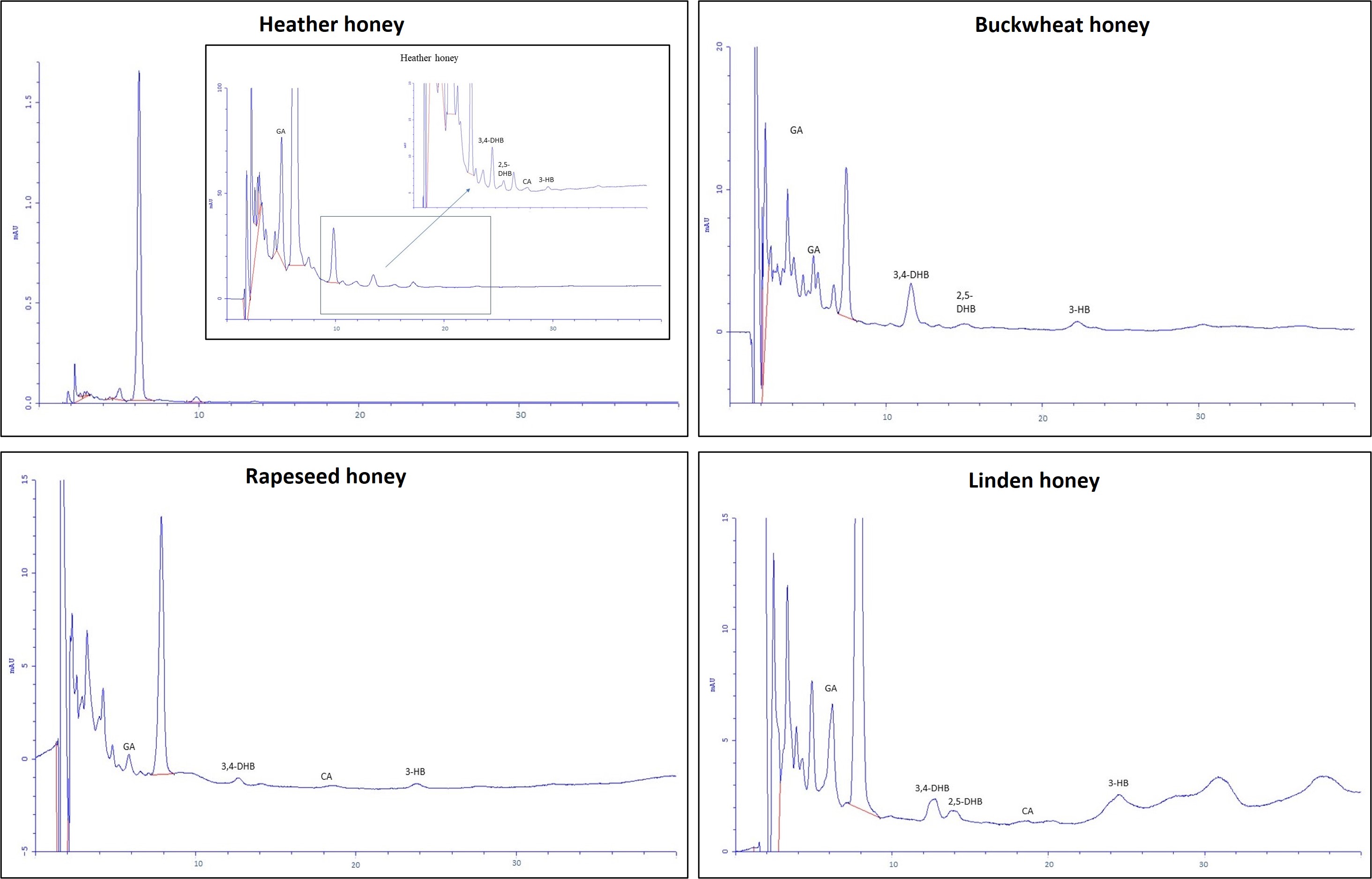

Supplement: Supplementary file 4 — Supplementary Material 4 [file 12906_2025_4786_MOESM4_ESM.jpg]

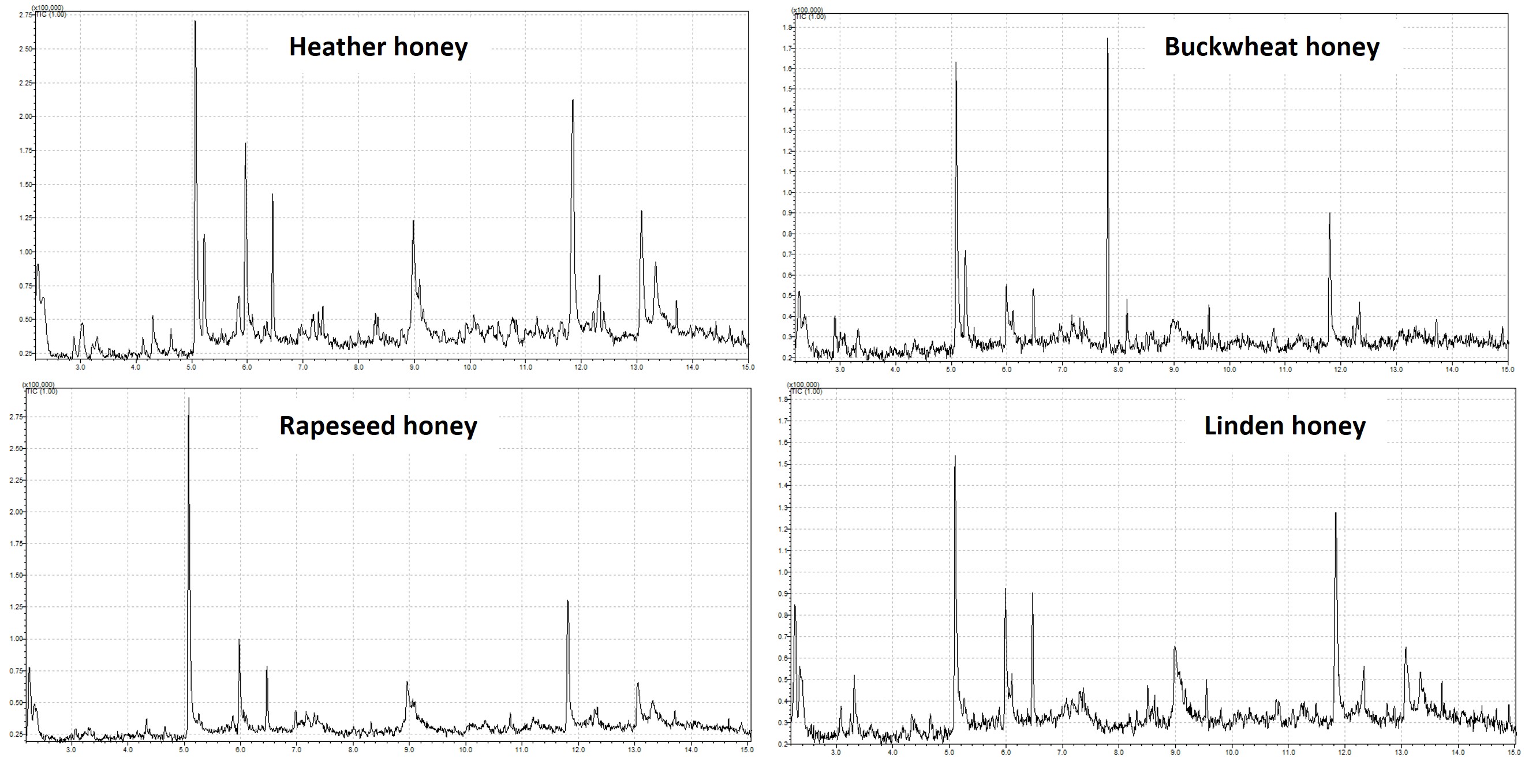

Supplement: Supplementary file 5 — Supplementary Material 5 [file 12906_2025_4786_MOESM5_ESM.jpg]

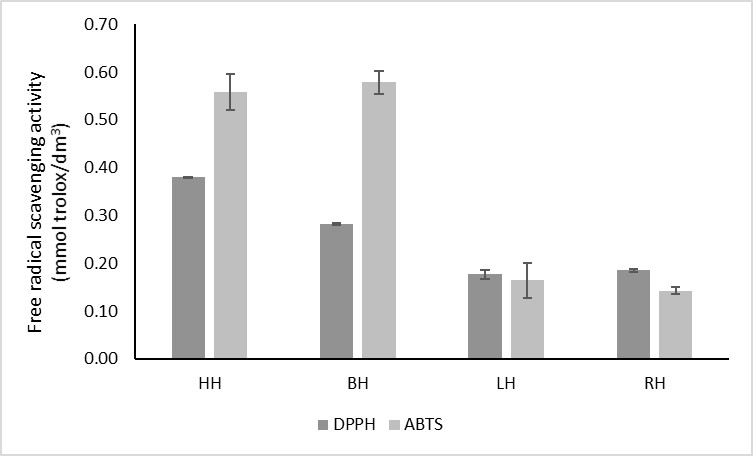

Supplement: Supplementary file 6 — Supplementary Material 6 [file 12906_2025_4786_MOESM6_ESM.jpg]

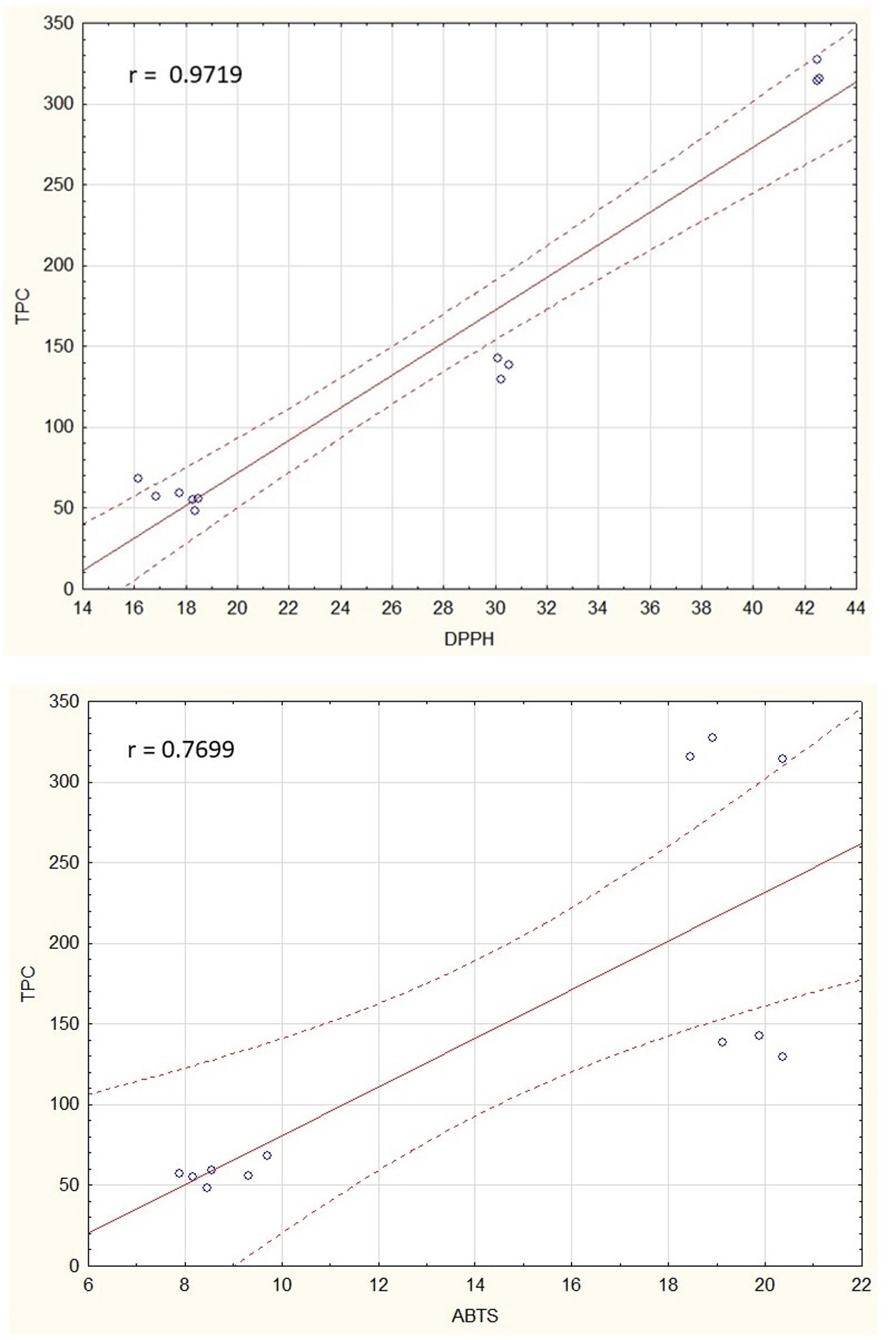

Supplement: Supplementary file 7 — Supplementary Material 7 [file 12906_2025_4786_MOESM7_ESM.jpg]

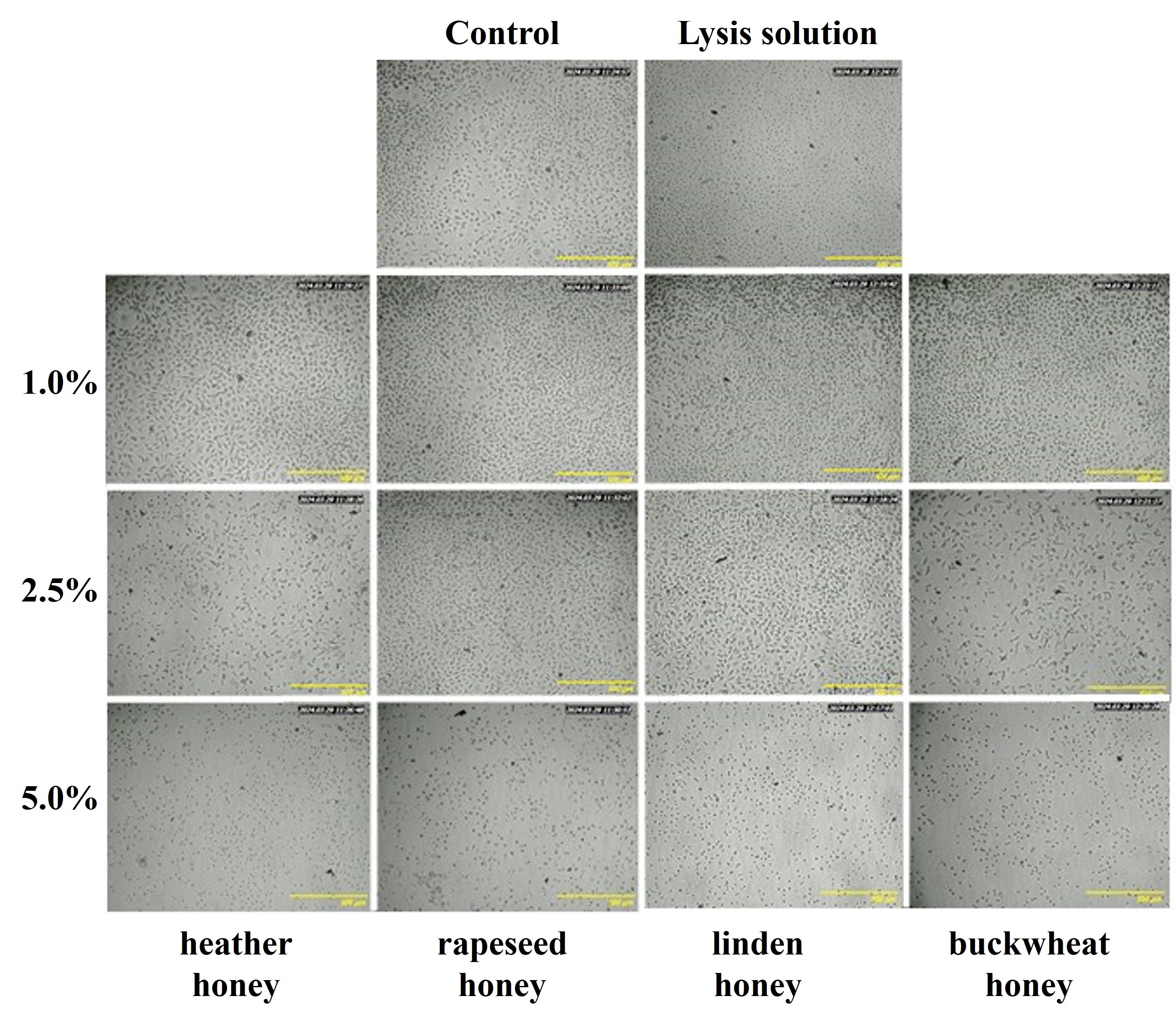

Supplement: Supplementary file 8 — Supplementary Material 8 [file 12906_2025_4786_MOESM8_ESM.jpg]
